# Supplementary material for: Synthesis and Biological Evaluation of Novel Allobetulon/Allobetulin–Nucleoside Conjugates as AntitumorAgents
Source: Molecules. 2022 Jul 25;27(15):4738. doi: 10.3390/molecules27154738 (PMC9329720; doi:10.3390/molecules27154738)
Supplement: Supplementary file 1 [file molecules-27-04738-s001.zip › molecules-1764143-supplementary.pdf]

Supplementary Materials

Synthesis and Biological Evaluation of Novel Allobetulon/Allobetulin–Nucleoside Conjugates as Antitumor Agents

Yanli Wang [1,2,†], Xiaowan Huang [3,4,†], Xiao Zhang [4], Jingchen Wang [4], Keyan Li [2], Guotao Liu [2], Kexin Lu [4], Xiang Zhang [3], Chengping Xie [3], Teresa Zheng [5], Yung-Yi Cheng [5,*] and Qiang Wang [1,2,3,4,*]

[1] School of Medicine, Huanghe Science and Technology College, Zhengzhou, China; hblyfe@163.com
[2] National Health Commission Key Laboratory of Birth Defect Prevention, Henan Institute of Reproductive Health Science and Technology, Zhengzhou, China; lky130277680782022@163.com (K.L.); liuguotao33@163.com (G.L.)
[3] High & New Technology Research Center, Henan Academy of Science, Zhengzhou, China; 18336708203@163.com (X.H.); zxzsy0405@163.com (X.Z.); xiechp@ceprei.com (C.X.)
[4] BGI College & Henan Institute of Medical and Pharmaceutical Science, Zhengzhou University, Zhengzhou, China; zhangxiao7011@163.com (X.Z.); chen13021083099@163.com (J.W.); kexin14590@163.com (K.L.)
[5] Division of Pharmacoengineering and Molecular Pharmaceutics, Eshelman School of Pharmacy, The University of North Carolina at Chapel Hill, Chapel Hill, NC, USA; tzheng1999@gmail.com
* Correspondence: yungyi@email.unc.edu (Y.-Y.C.); qiangwang@hhstu.edu.cn (Q.W.); Tel: +1-9843694796 (Y.-Y.C.); +86-371-65511776 (Q.W.)
† These authors contributed equally to this work.

Figure S1 ^{1}H NMR spectrum of compound 5....4
FigureS2 ^{13}C NMR spectrum of compound 5....4
Figure S3 HRMS spectrum of compound 5....5
Figure S4 ^{1}H NMR spectrum of compound 6....5
Figure S5 ^{13}C NMR spectrum of compound 6....6
Figure S6 HRMS spectrum of compound 6....6
Figure S7 ^{1}H NMR spectrum of compound 7....7
Figure S8 ^{13}C NMR spectrum of compound 7....7
Figure S9 HRMS spectrum of compound 7....8
Figure S10 ^{1}H NMR spectrum of compound 8....8
Figure S11 Expanded NOESY spectrum (0.6-2.0 ppm) of compound 8....9
Figure S12 Expanded COSY spectrum (0.6-2.0 ppm) of compound 8....9
Figure S13 ^{13}C NMR spectrum of compound 8....10
Figure S14 HRMS spectrum of compound 8....10
Figure S15 ^{1}H NMR spectrum of compound 9a....11
Figure S16 ^{13}C NMR spectrum of compound 9a....11

Figure S17 HRMS spectrum of compound 9a..12
Figure S18 ^{1}H NMR spectrum of compound 9b..12
Figure S19 ^{13}C NMR spectrum of compound 9b...13
Figure S20 HRMS spectrum of compound 9b..13
Figure S21 ^{1}H NMR spectrum of compound 9c..14
Figure S22 ^{13}C NMR spectrum of compound 9c...14
Figure S23 HRMS spectrum of compound 9c..15
Figure S24 ^{1}H NMR spectrum of compound 9d..15
Figure S25 ^{13}C NMR spectrum of compound 9d...16
Figure S26 HRMS spectrum of compound 9d..16
Figure S27 ^{1}H NMR spectrum of compound 9e..17
Figure S28 ^{13}C NMR spectrum of compound 9e...17
Figure S29 HRMS spectrum of compound 9e..18
Figure S30 ^{1}H NMR spectrum of compound 9f..18
Figure S31 ^{13}C NMR spectrum of compound 9f...19
Figure S32 HRMS spectrum of compound 9f..19
Figure S33 ^{1}H NMR spectrum of compound 9g..20
Figure S34 ^{13}C NMR spectrum of compound 9g...20
Figure S35 HRMS spectrum of compound 9g..21
Figure S36 ^{1}H NMR spectrum of compound 9h..21
Figure S37 ^{13}C NMR spectrum of compound 9h...22
Figure S38 HRMS spectrum of compound 9h..22
Figure S39 ^{1}H NMR spectrum of compound 9i..23
Figure S40 ^{13}C NMR spectrum of compound 9i...23
Figure S41 HRMS spectrum of compound 9i..24
Figure S42 ^{1}H NMR spectrum of compound 10a..24
Figure S43 ^{13}C NMR spectrum of compound 10a...25
Figure S44 HRMS spectrum of compound 10a..25
Figure S45 ^{1}H NMR spectrum of compound 10b..26
Figure S46 ^{13}C NMR spectrum of compound 10b...26
Figure S47 HRMS spectrum of compound 10b....................... 27
Figure S48 ^{1}H NMR spectrum of compound 10c..27
Figure S49 ^{13}C NMR spectrum of compound 10c..28
Figure S50 HRMS spectrum of compound 10c..28

Figure S51 ^{1}H NMR spectrum of compound 10d..29
Figure S52 ^{13}C NMR spectrum of compound 10d..29
Figure S53 HRMS spectrum of compound 10d..30
Figure S54 ^{1}H NMR spectrum of compound 10e..30
Figure S55 ^{13}C NMR spectrum of compound 10e...31
Figure S56 HRMS spectrum of compound 10e...31
Figure S57 ^{1}H NMR spectrum of compound 10f...32
Figure S58 ^{13}C NMR spectrum of compound 10f..32
Figure S59 HRMS spectrum of compound 10f..33
Figure S60 ^{1}H NMR spectrum of compound 10g..33
Figure S61 ^{13}C NMR spectrum of compound 10g..34
Figure S62 HRMS spectrum of compound 10g...34
Figure S63 ^{1}H NMR spectrum of compound 10h..35
Figure S64 ^{13}C NMR spectrum of compound 10h...35
Figure S65 HRMS spectrum of compound 10h...36
Figure S66 ^{1}H NMR spectrum of compound 10i...36
Figure S67 ^{13}C NMR spectrum of compound 10i..37
Figure S68 HRMS spectrum of compound 10i..37

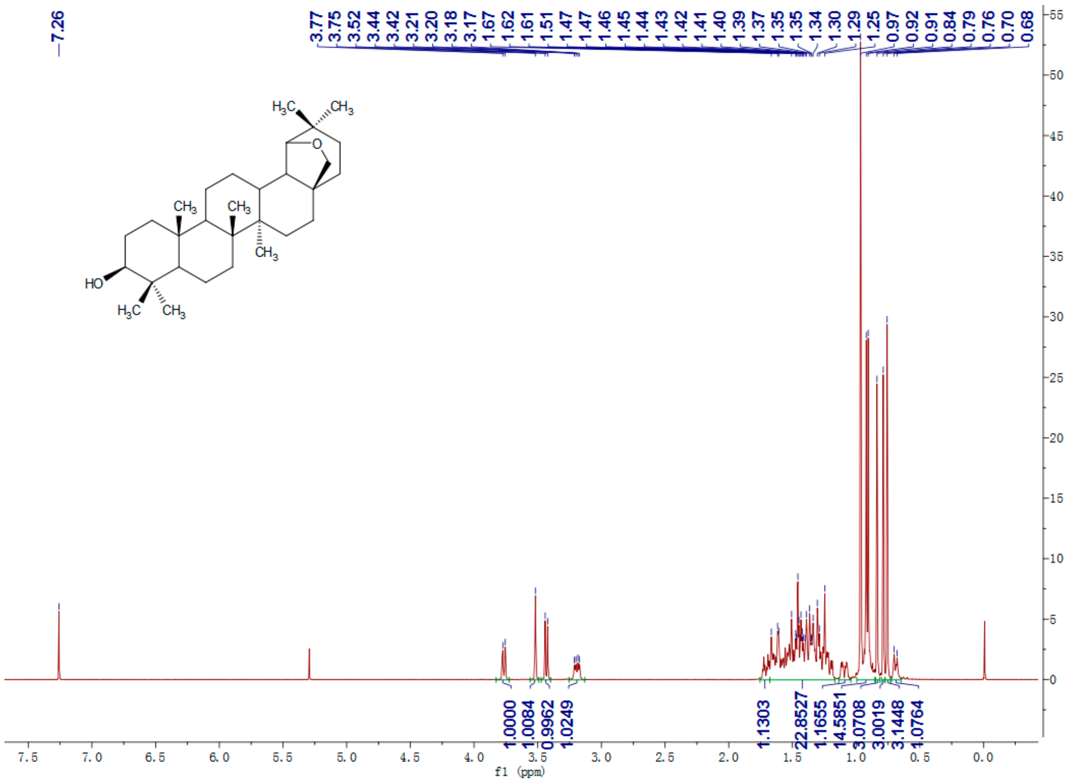

Figure S1 ^{1}H NMR spectrum of compound **5**

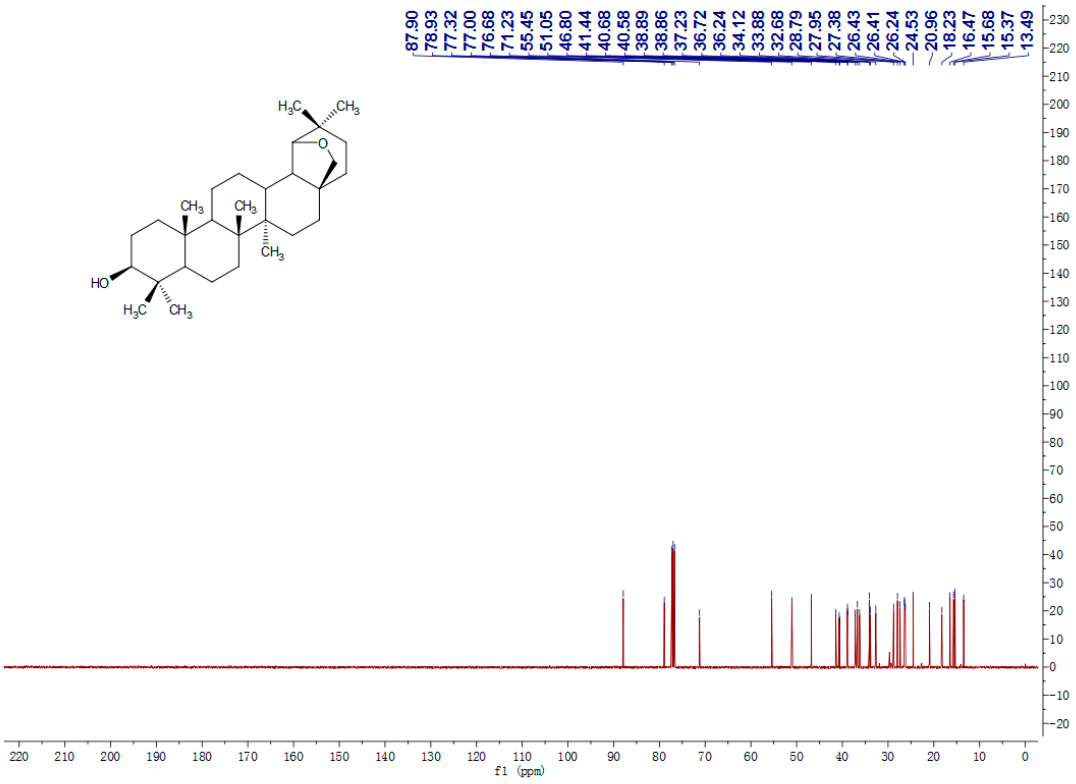

Figure S2 ^{13}C NMR spectrum of compound **5**

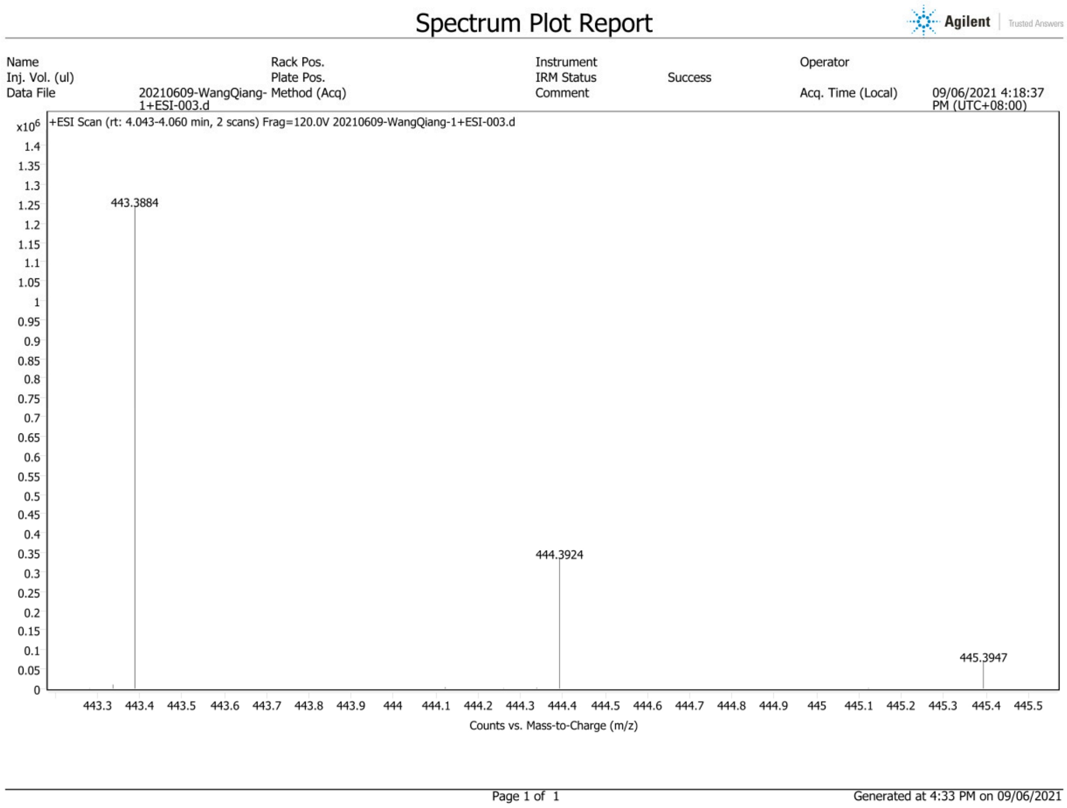

Figure S3 HRMS spectrum of compound **5**

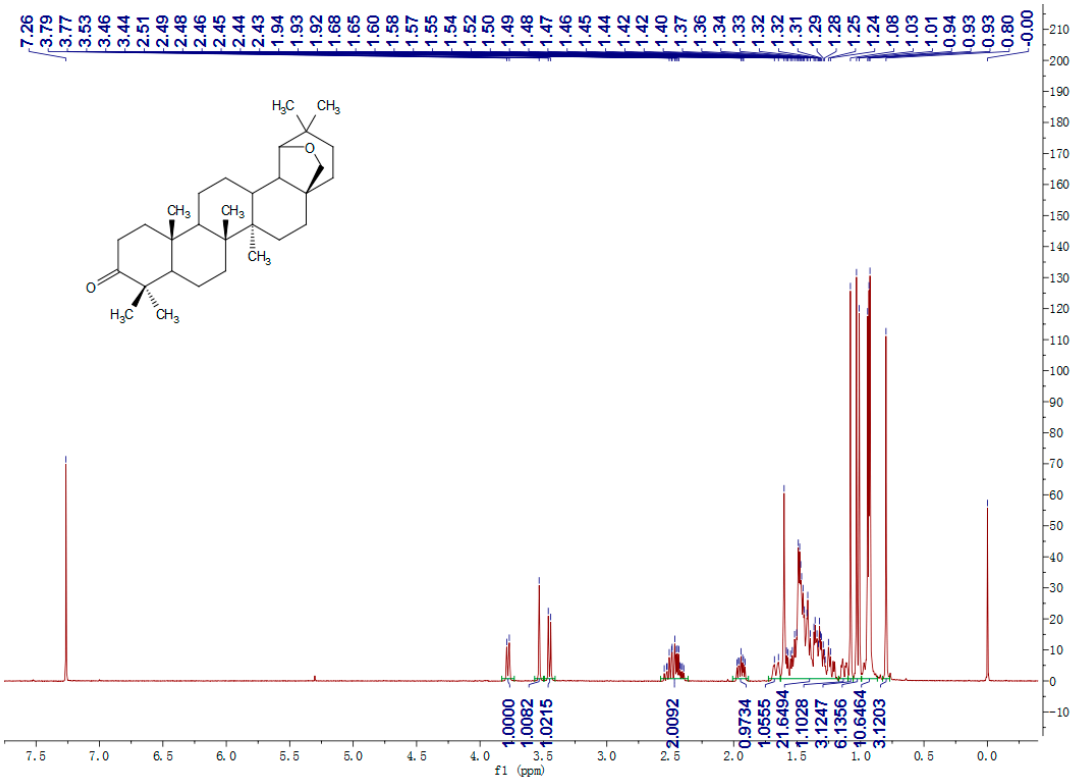

Figure S4 ^{1}H NMR spectrum of compound **6**

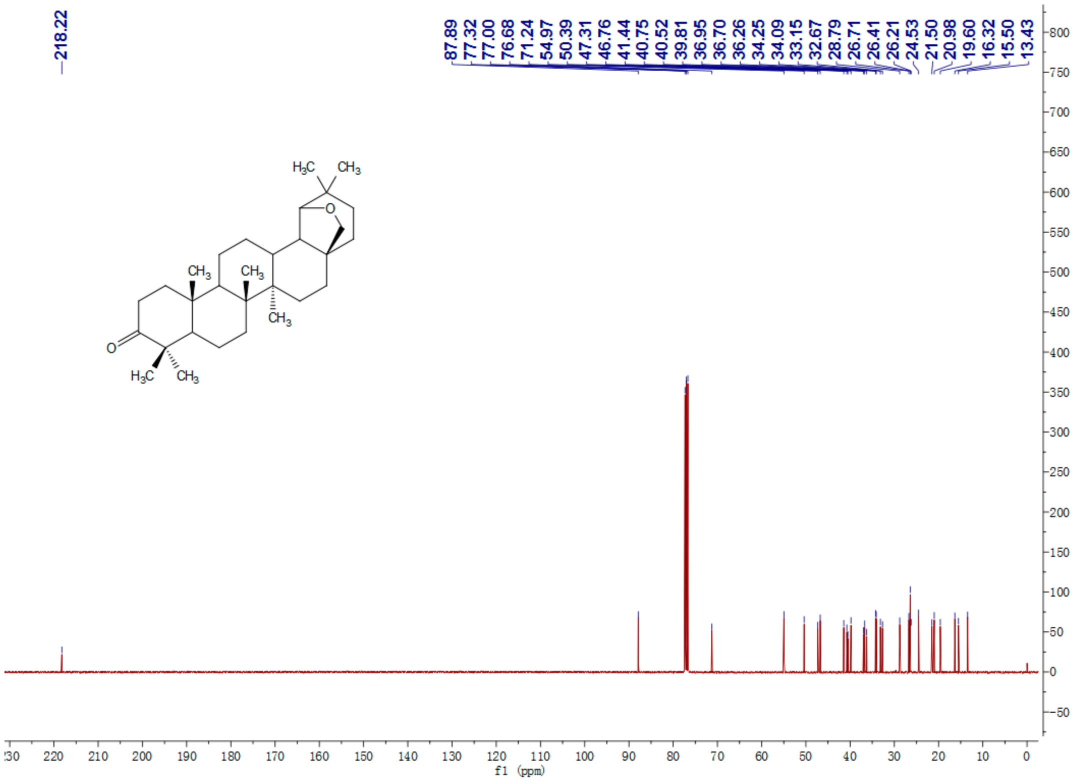

Figure S5 ^{13}C NMR spectrum of compound **6**

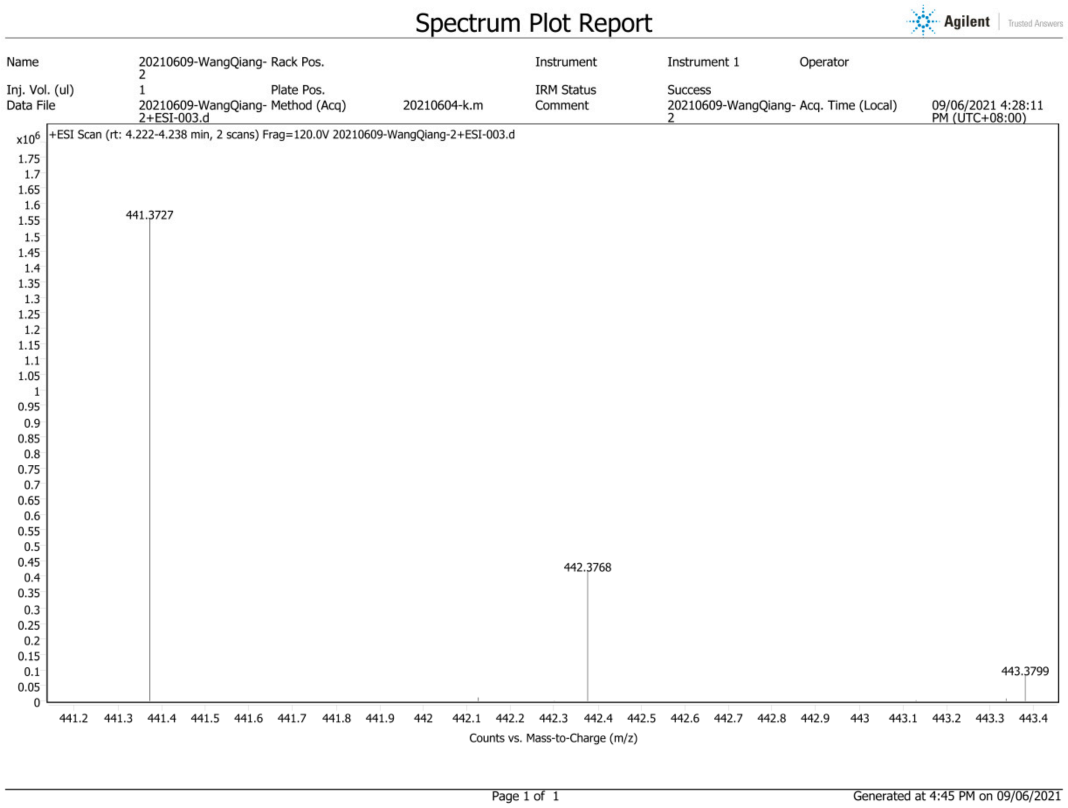

Figure S6 HRMS spectrum of compound **6**

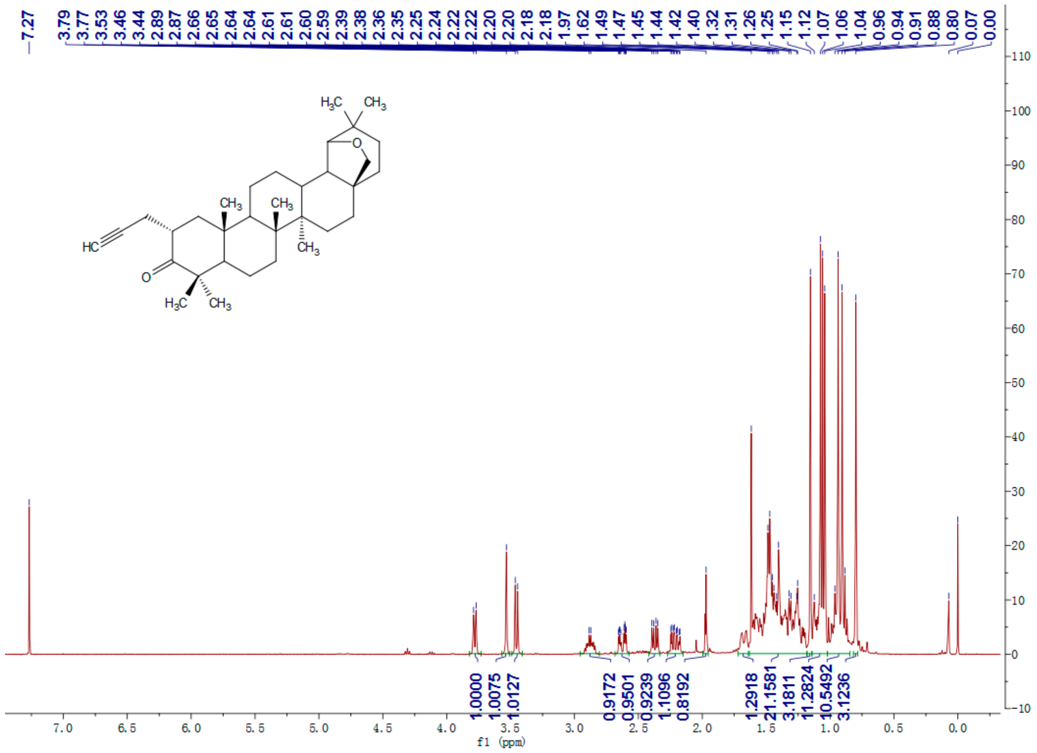

Figure S7 ^{1}H NMR spectrum of compound **7**

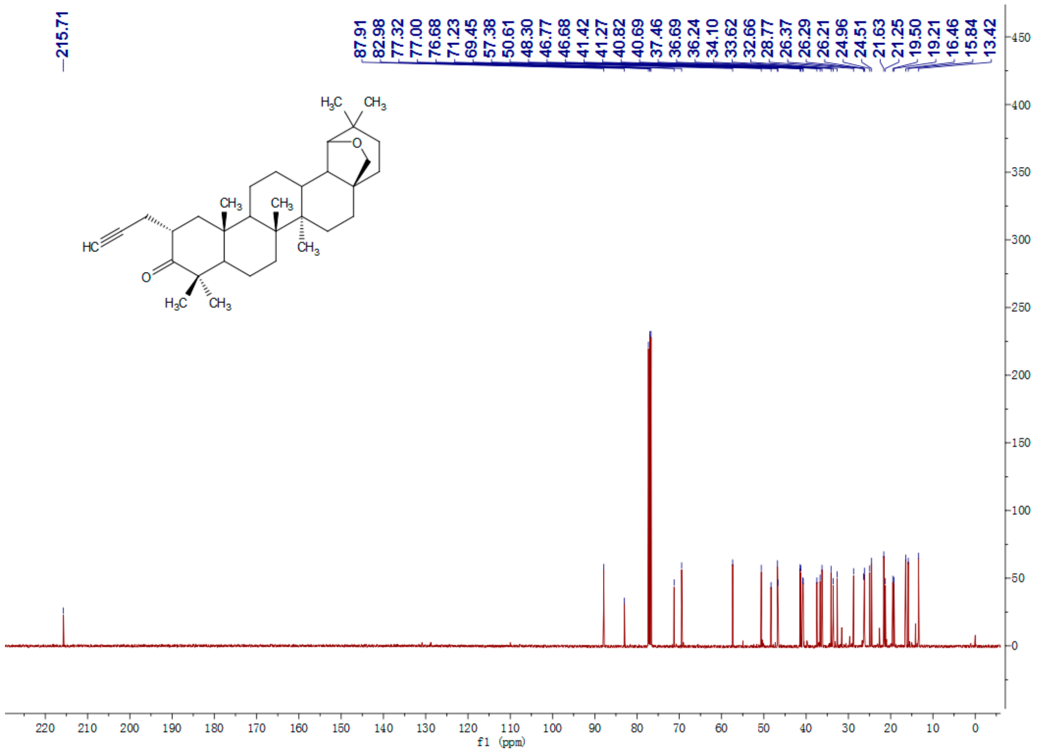

Figure S8 ^{13}C NMR spectrum of compound **7**

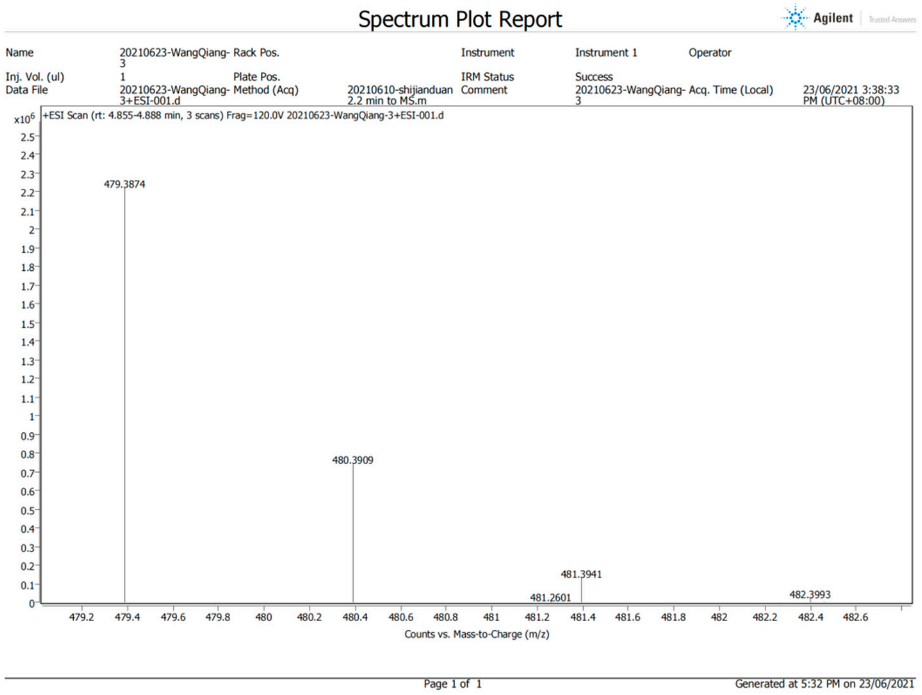

Figure S9 HRMS spectrum of compound **7**

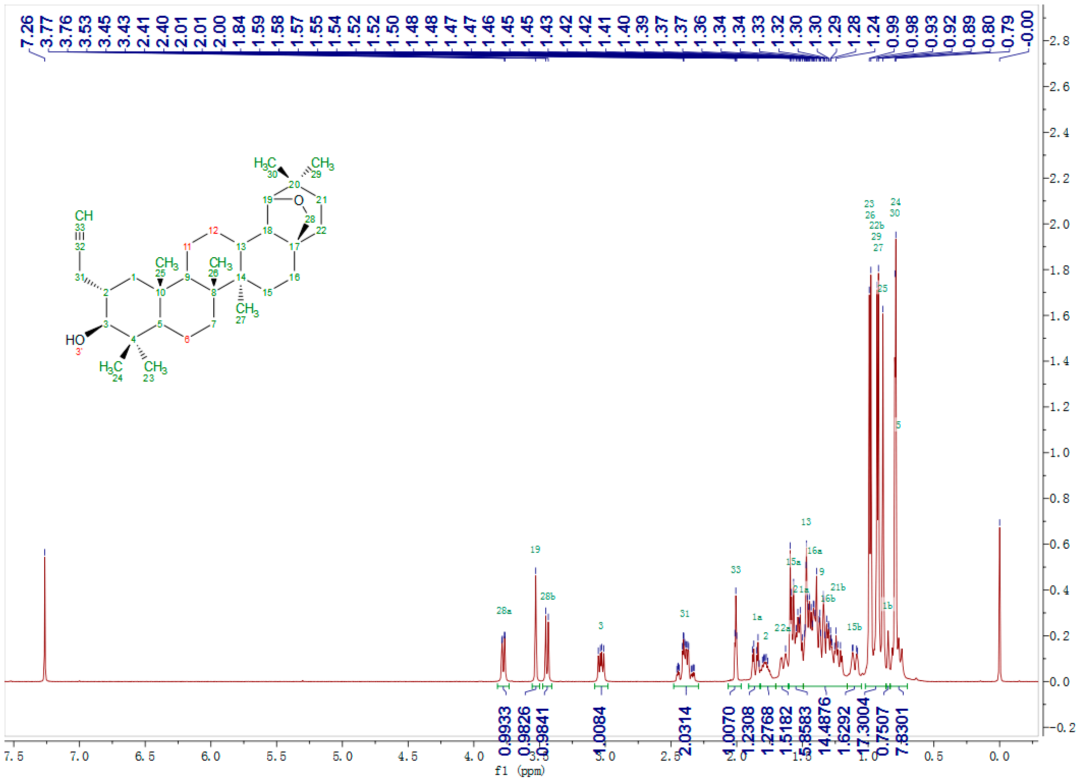

Figure S10 ^{1}H NMR spectrum of compound **8**

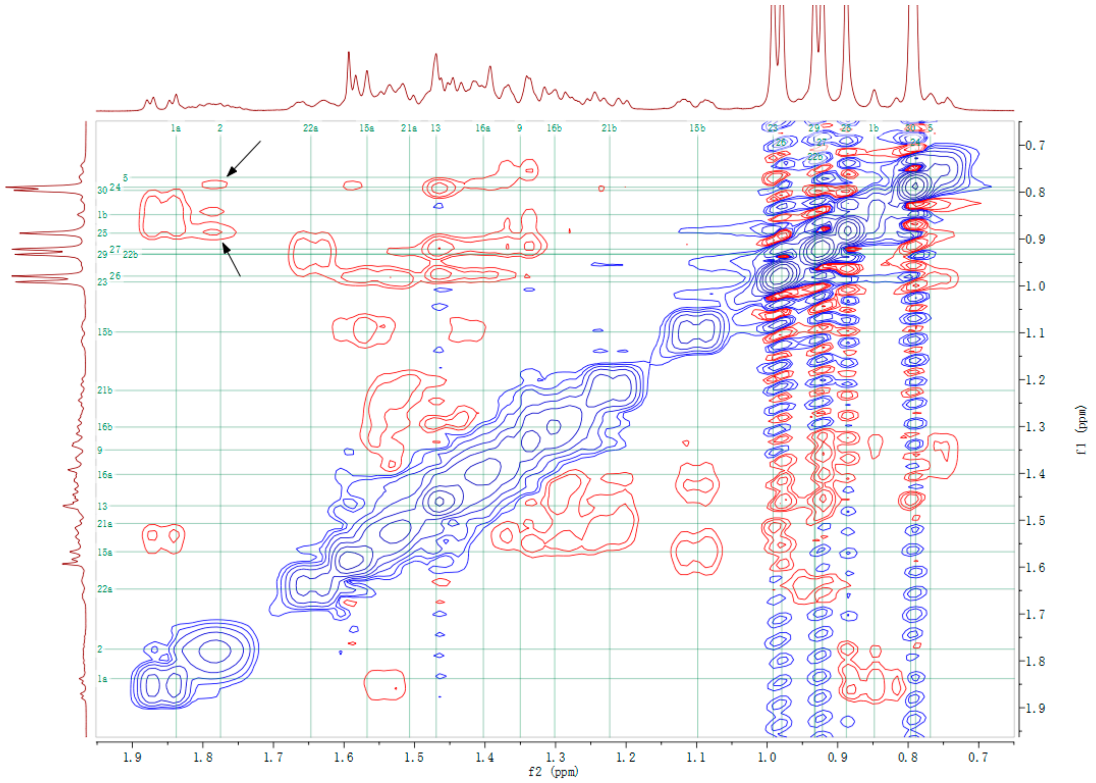

Figure S11 Expanded NOESY spectrum (0.6-2.0 ppm) of compound **8**

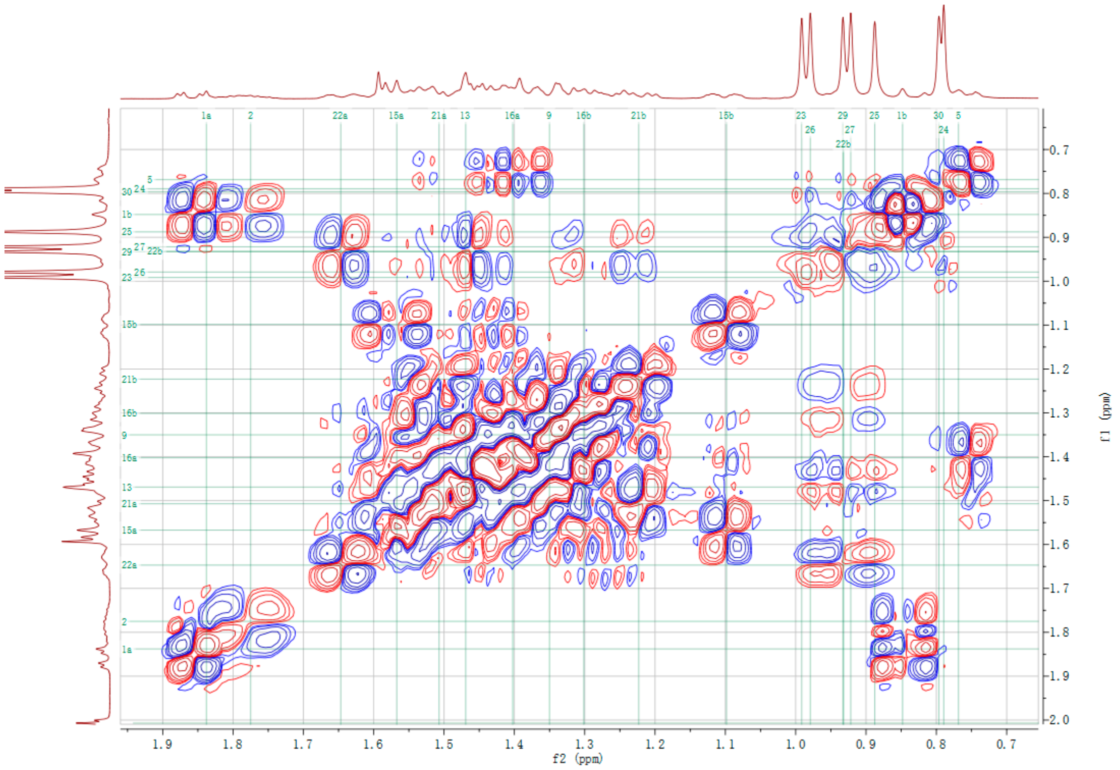

Figure S12 Expanded COSY spectrum (0.6-2.0 ppm) of compound **8**

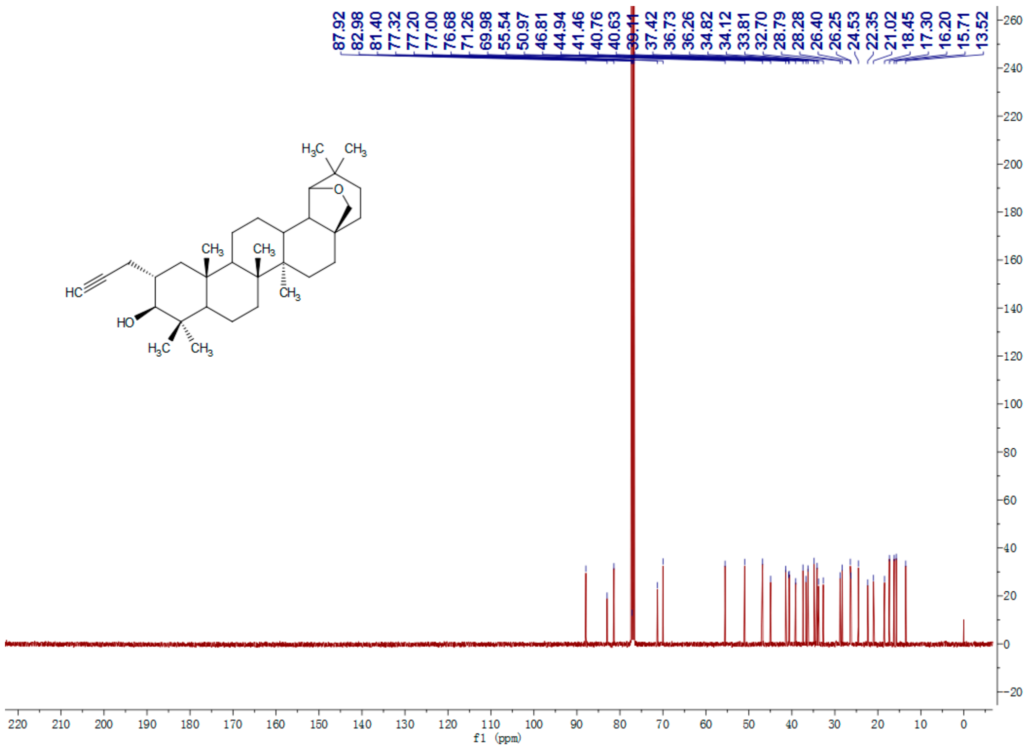

Figure S13 ^{13}C NMR spectrum of compound **8**

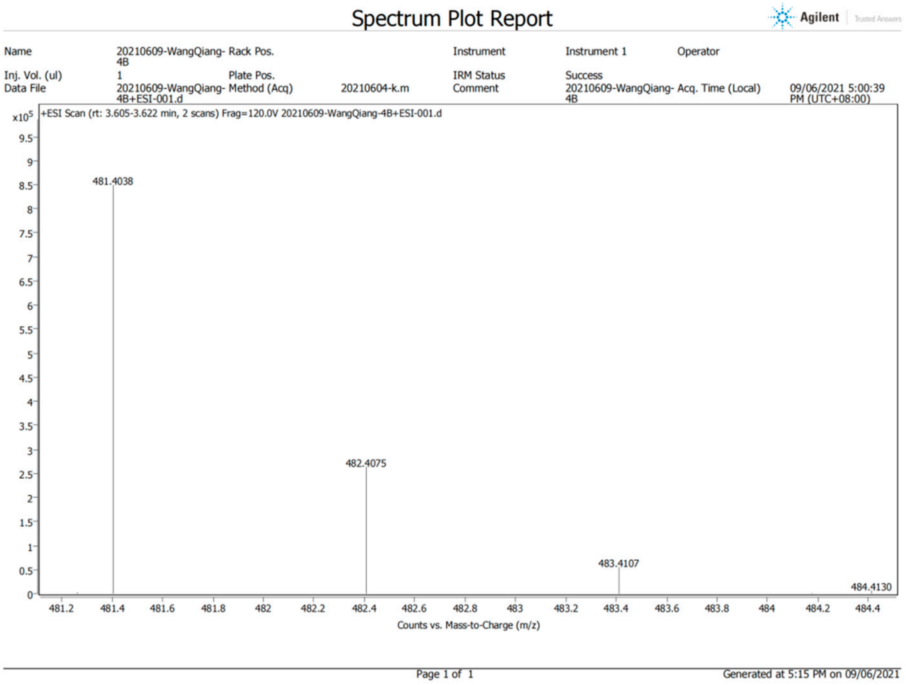

Figure S14 HRMS spectrum of compound **8**

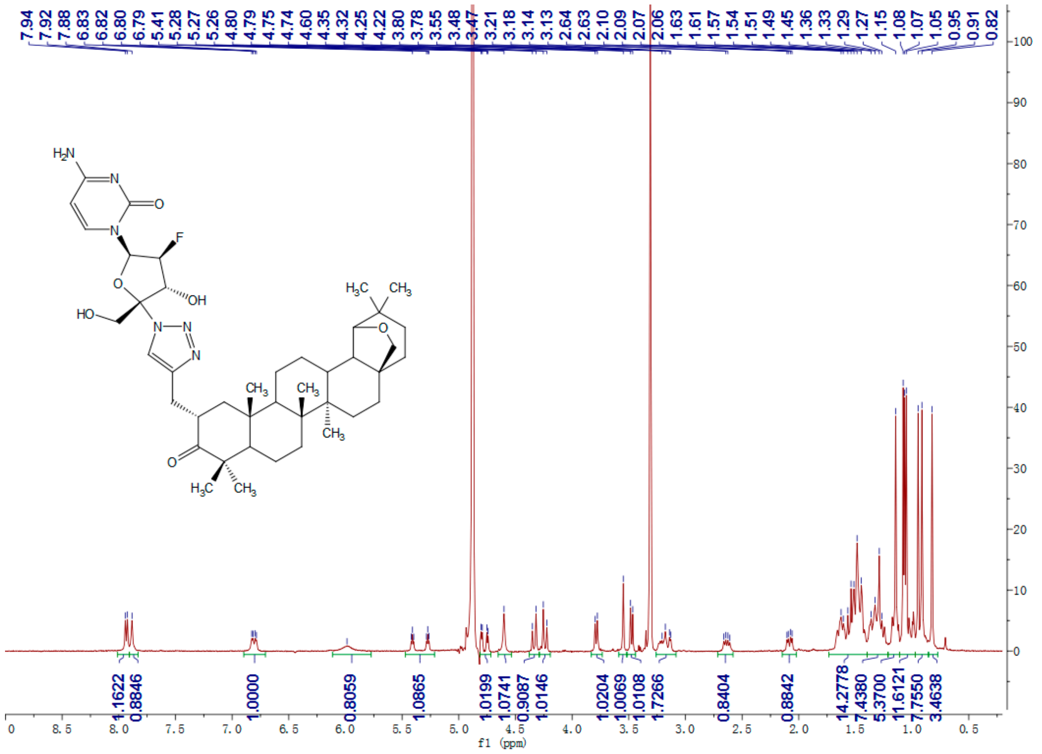

Figure S15 ^{1}H NMR spectrum of compound **9a**

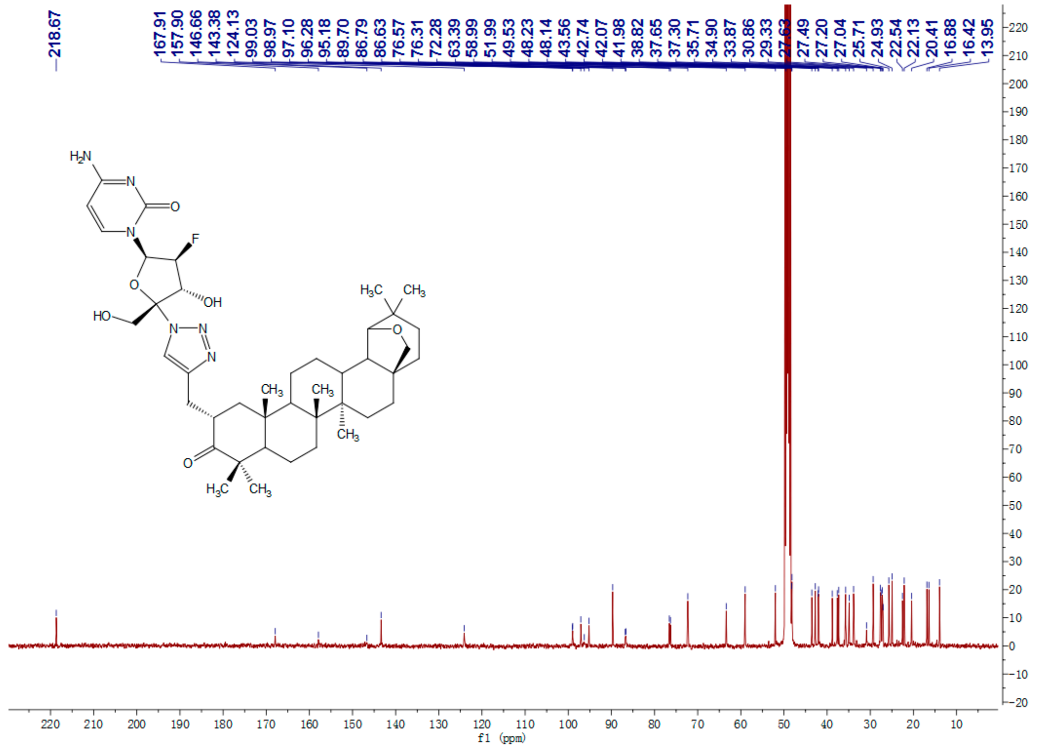

Figure S16 ^{13}C NMR spectrum of compound **9a**

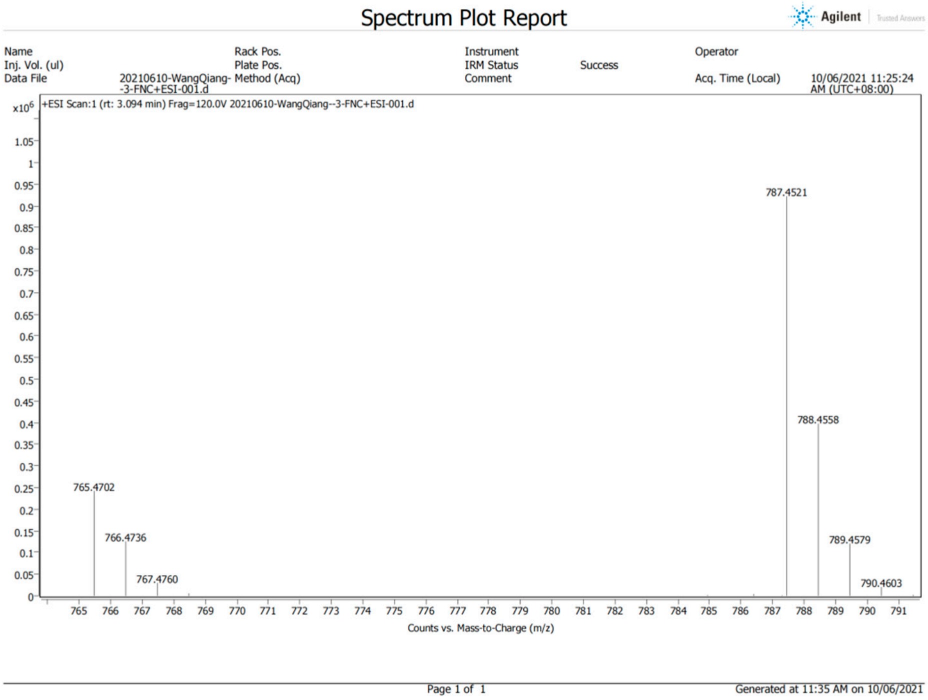

Figure S17 HRMS spectrum of compound **9a**

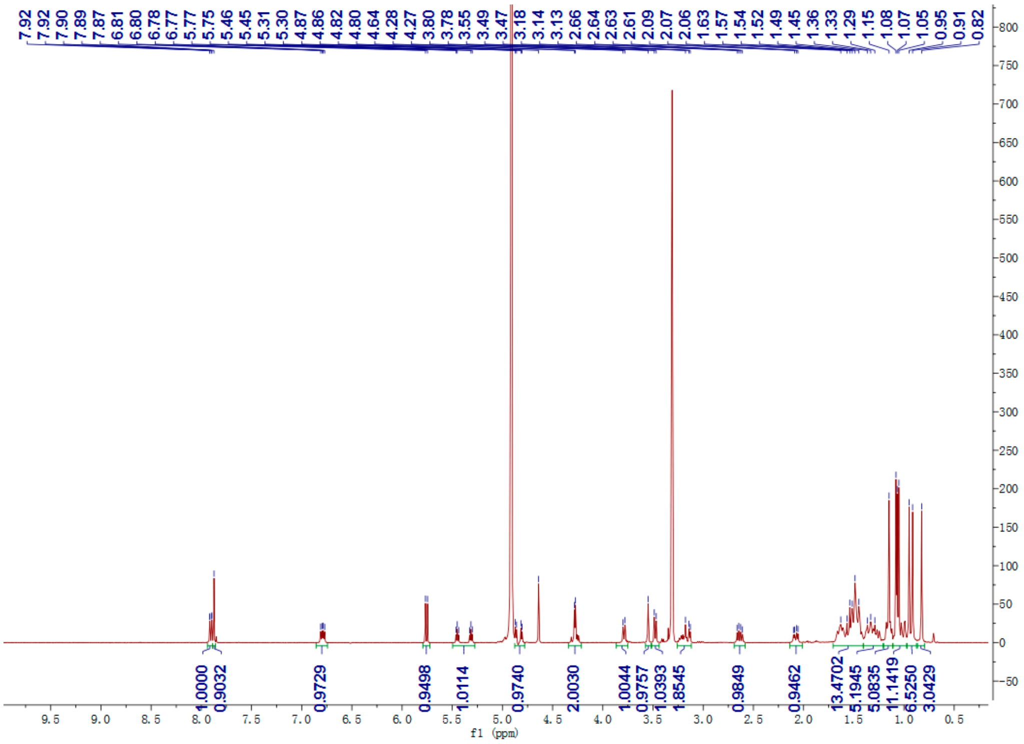

Figure S18 ^{1}H NMR spectrum of compound **9b**

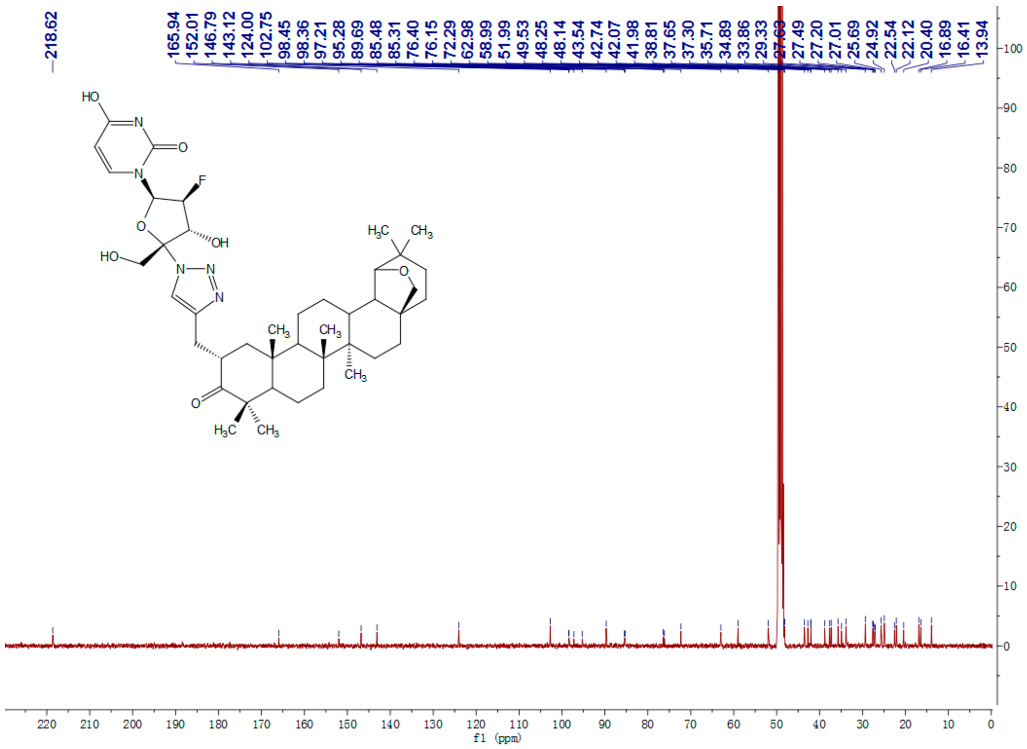

Figure S19 ^{13}C NMR spectrum of compound **9b**

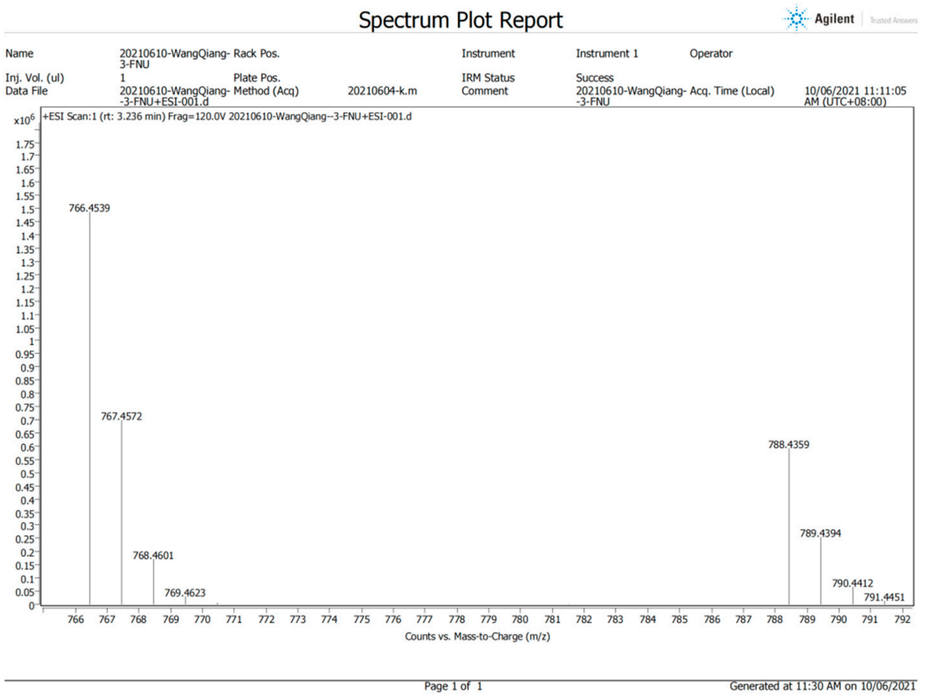

Figure S20 HRMS spectrum of compound **9b**

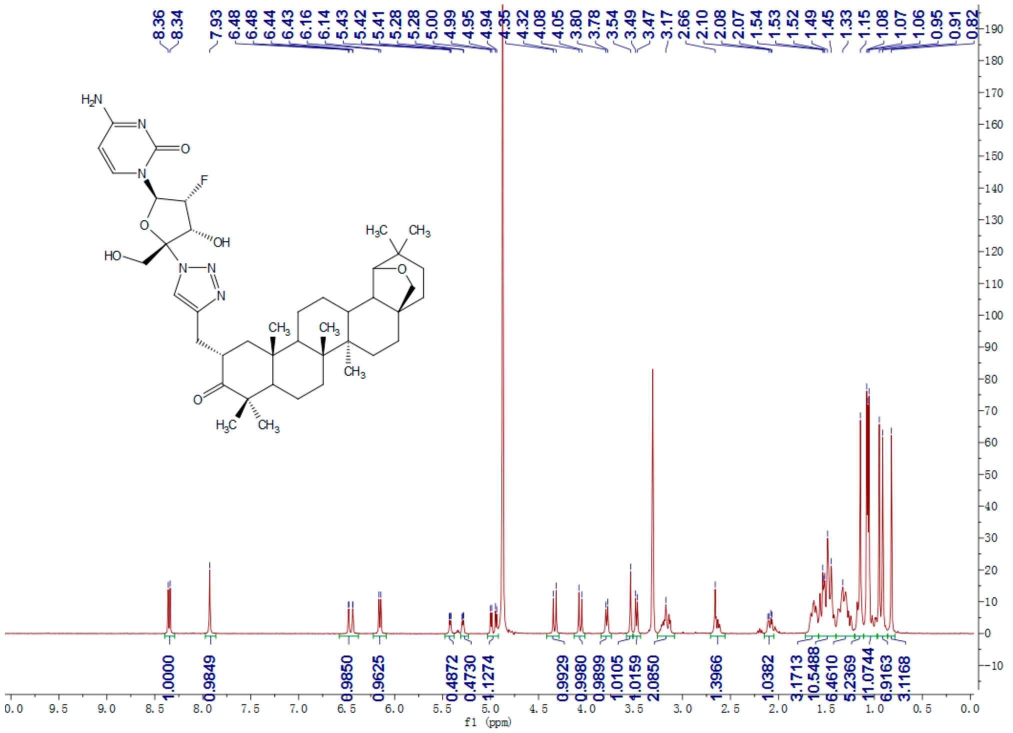

Figure S21 ^{1}H NMR spectrum of compound **9c**

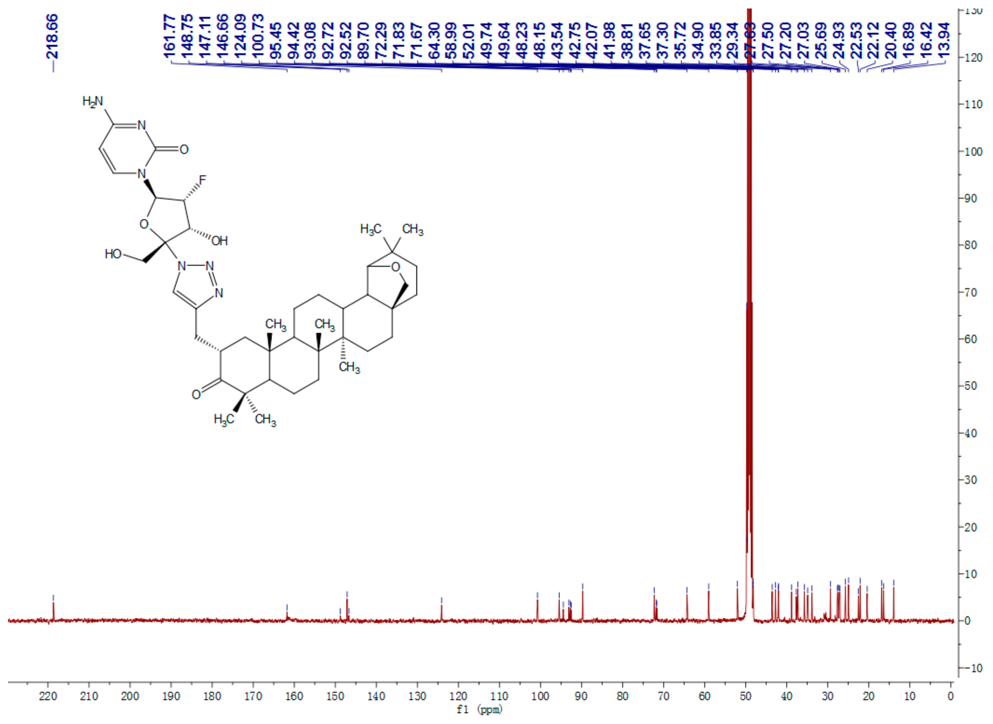

Figure S22 ^{13}C NMR spectrum of compound **9c**

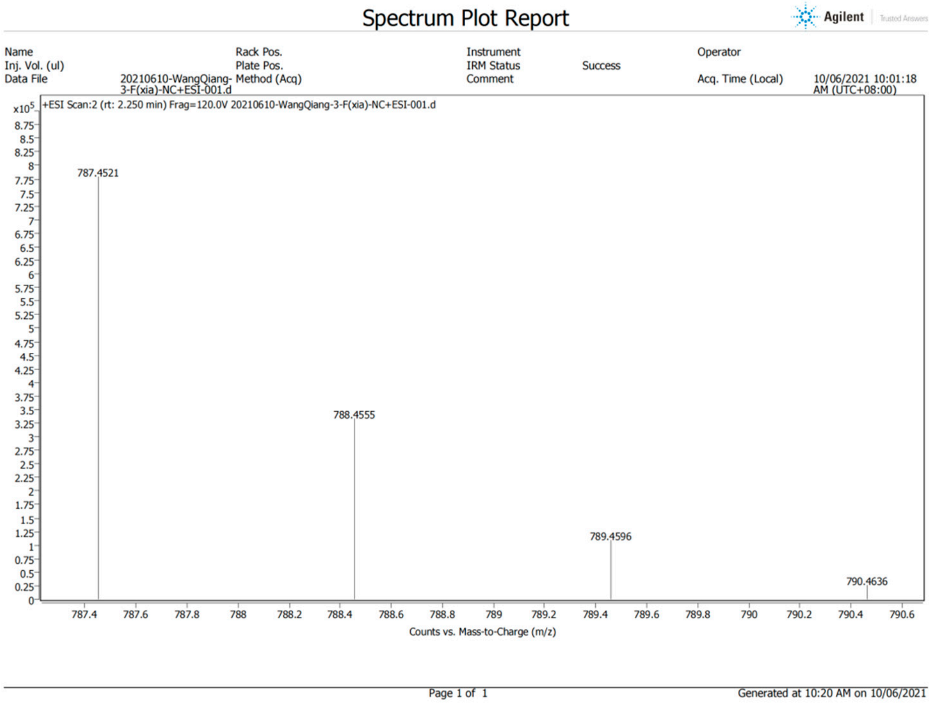

Figure S23 HRMS spectrum of compound **9c**

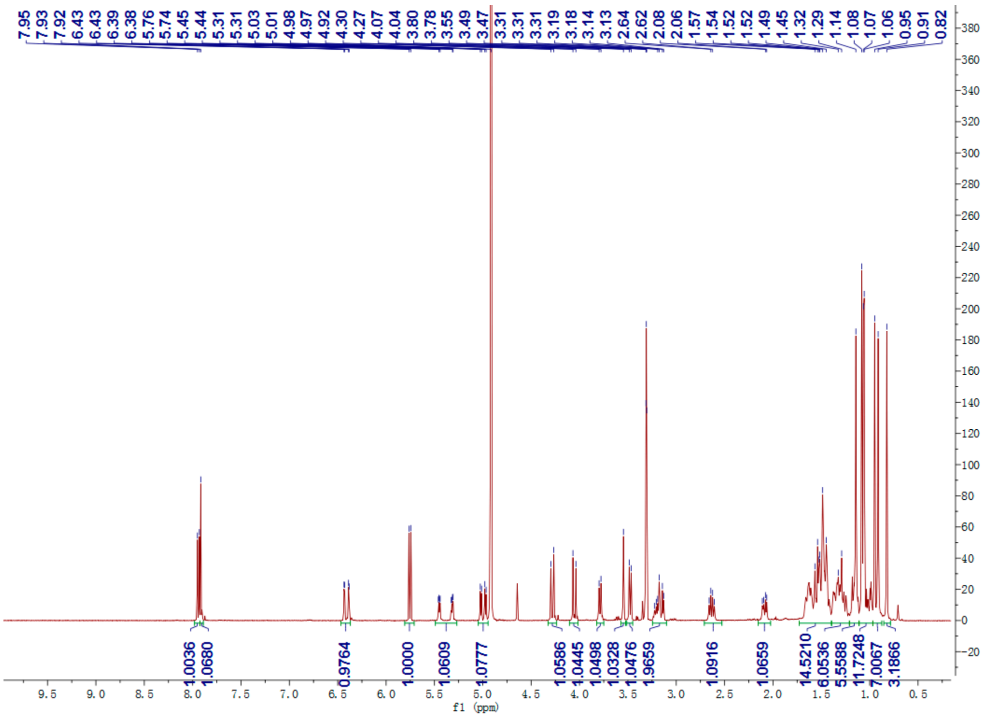

Figure S24 ^{1}H NMR spectrum of compound **9d**

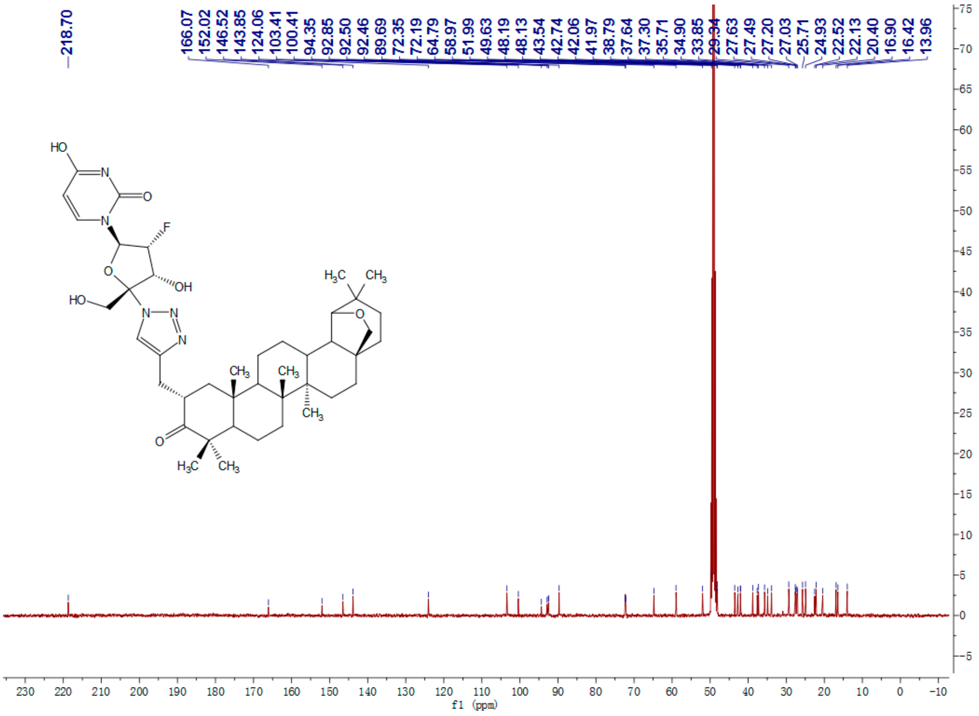

Figure S25 ^{13}C NMR spectrum of compound **9d**

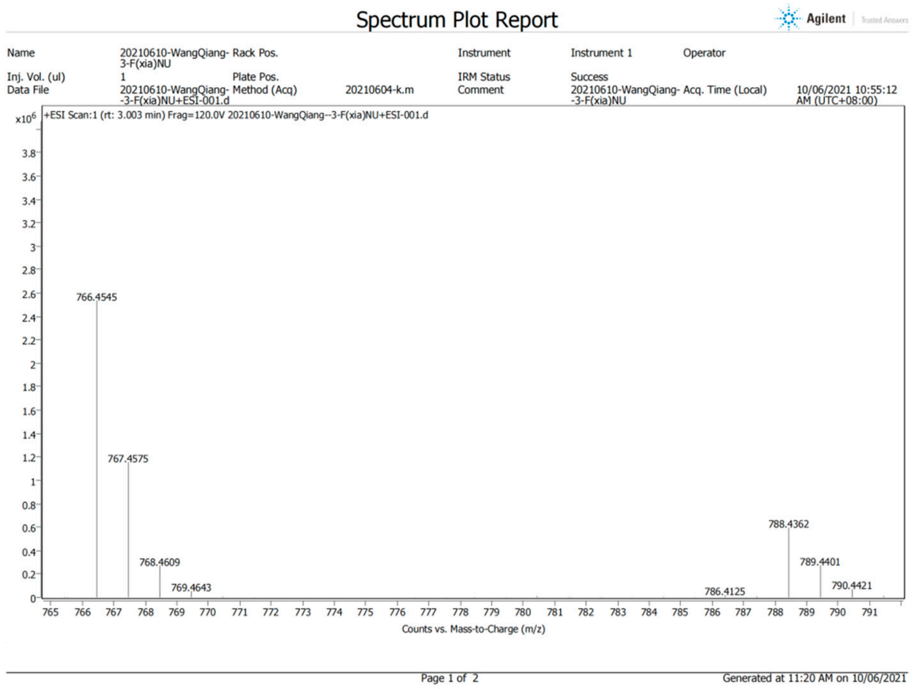

Figure S26 HRMS spectrum of compound **9d**

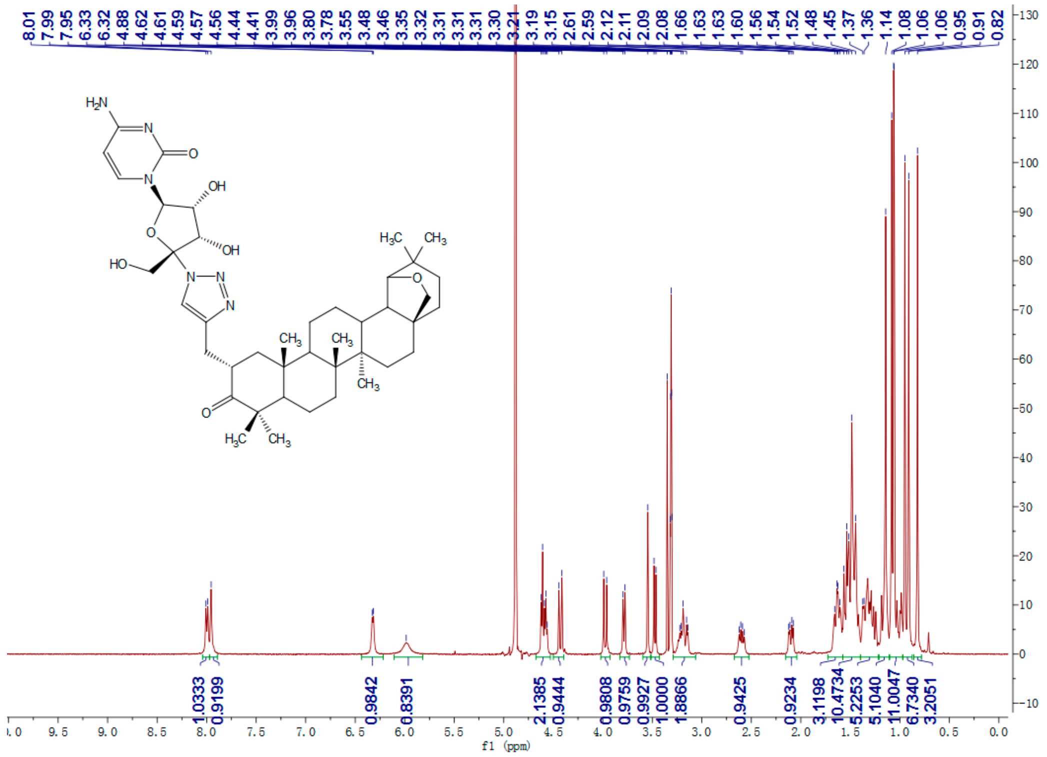

Figure S27 ^{1}H NMR spectrum of compound **9e**

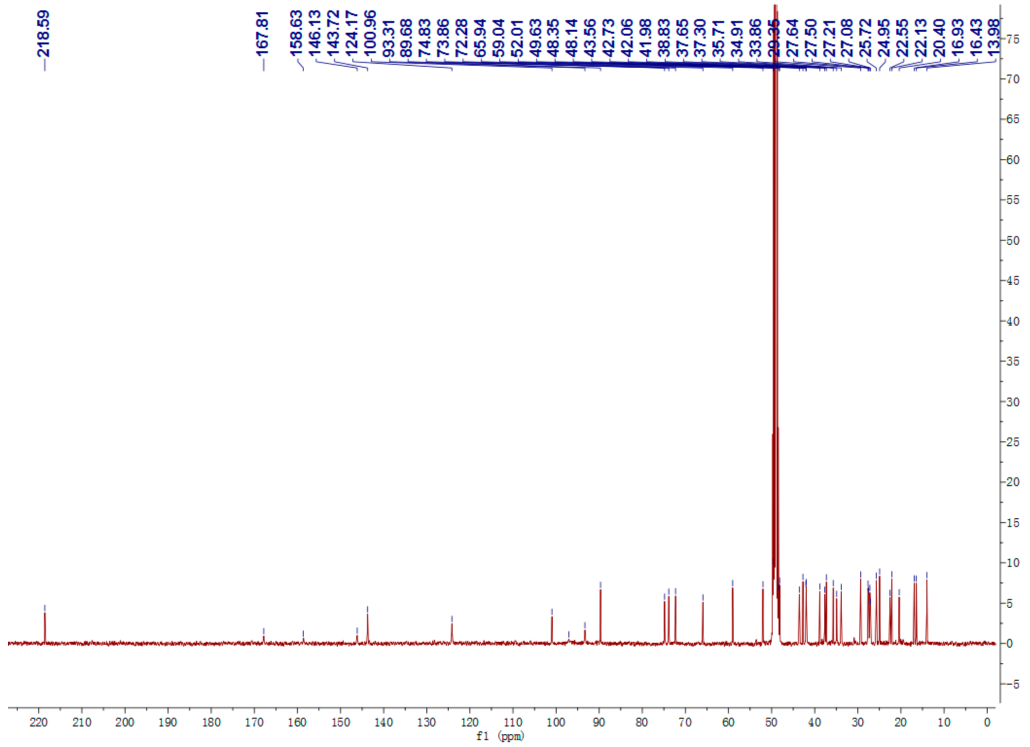

Figure S28 ^{13}C NMR spectrum of compound **9e**

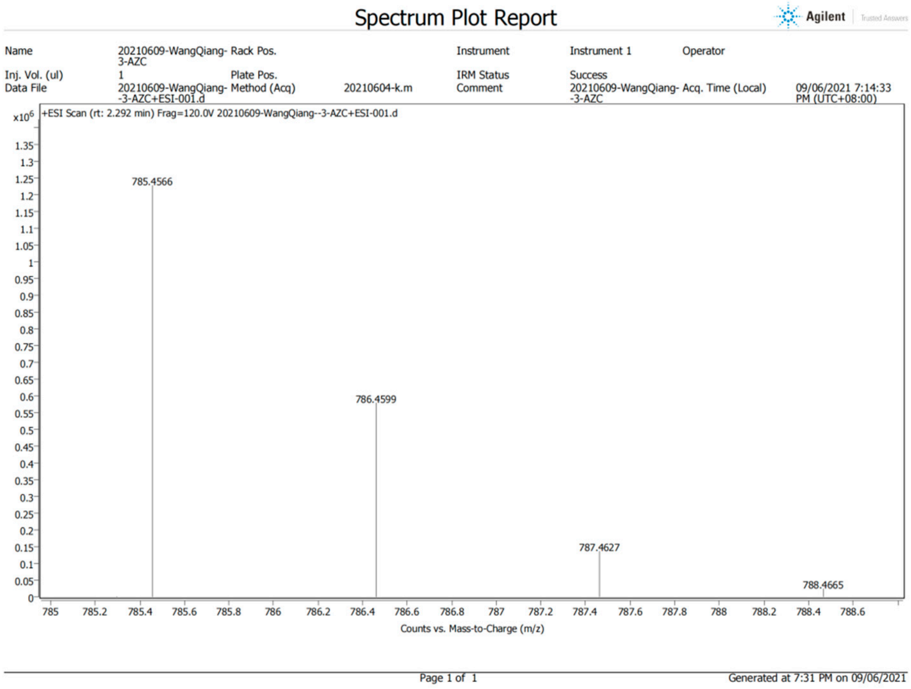

Figure S29 HRMS spectrum of compound **9e**

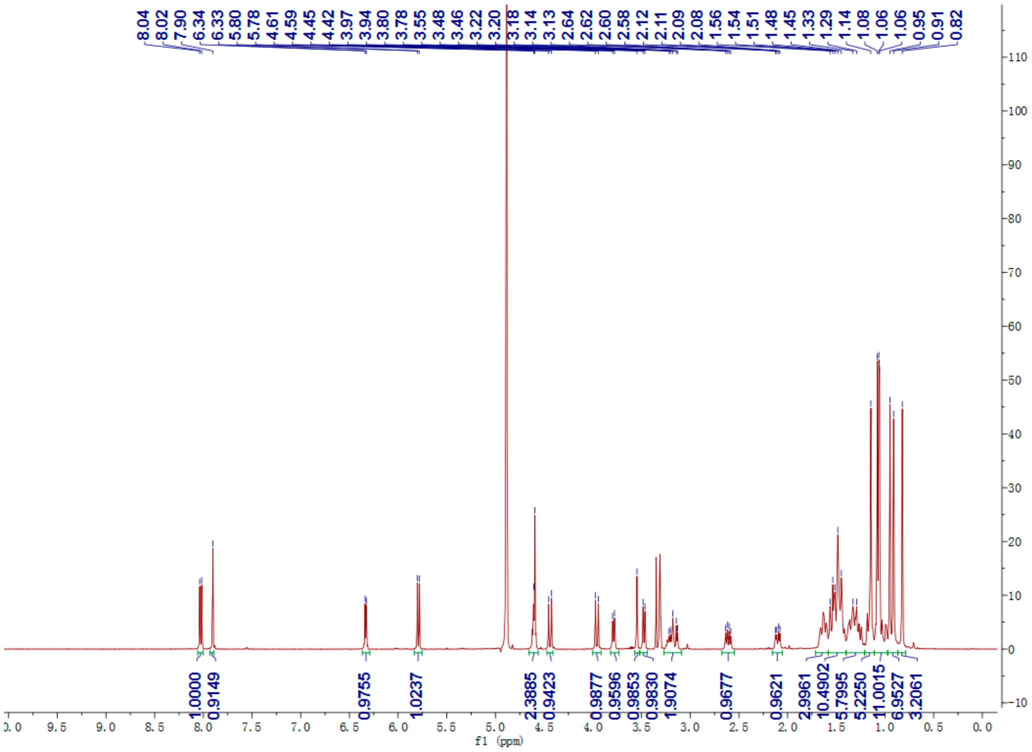

Figure S30 ^{1}H NMR spectrum of compound **9f**

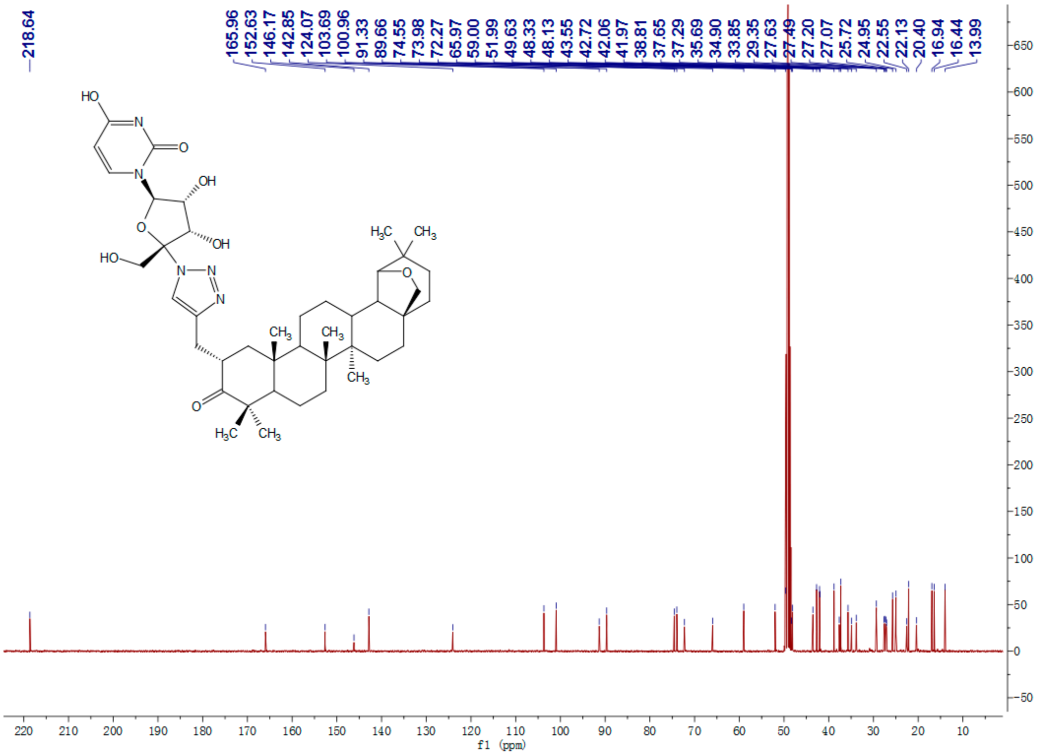

Figure S31 ^{13}C NMR spectrum of compound **9f**

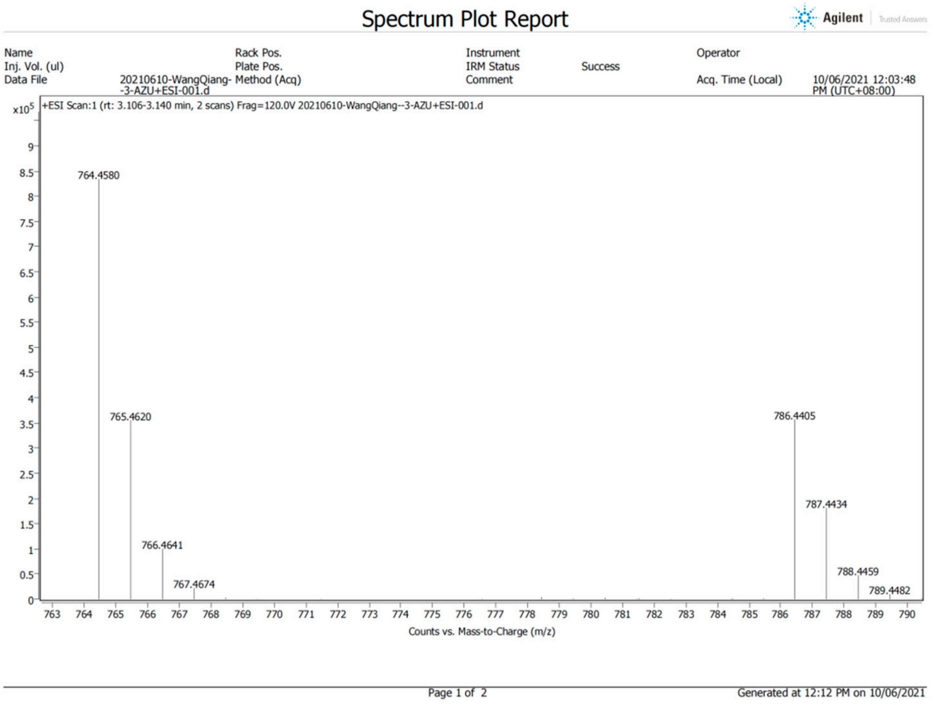

Figure S32 HRMS spectrum of compound **9f**

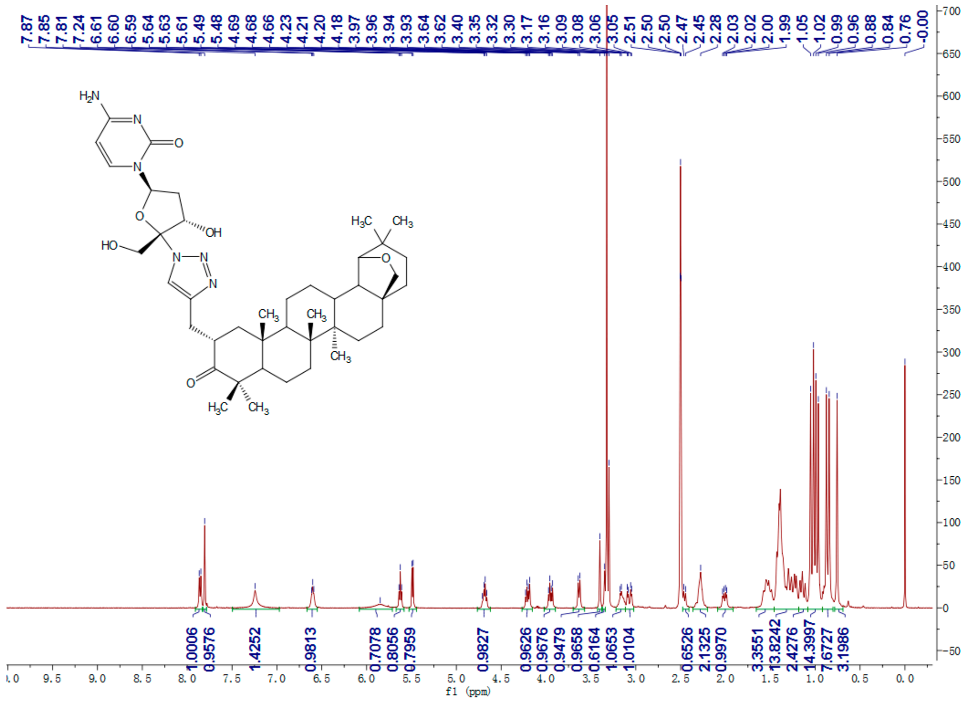

Figure S33 ^{1}H NMR spectrum of compound **9g**

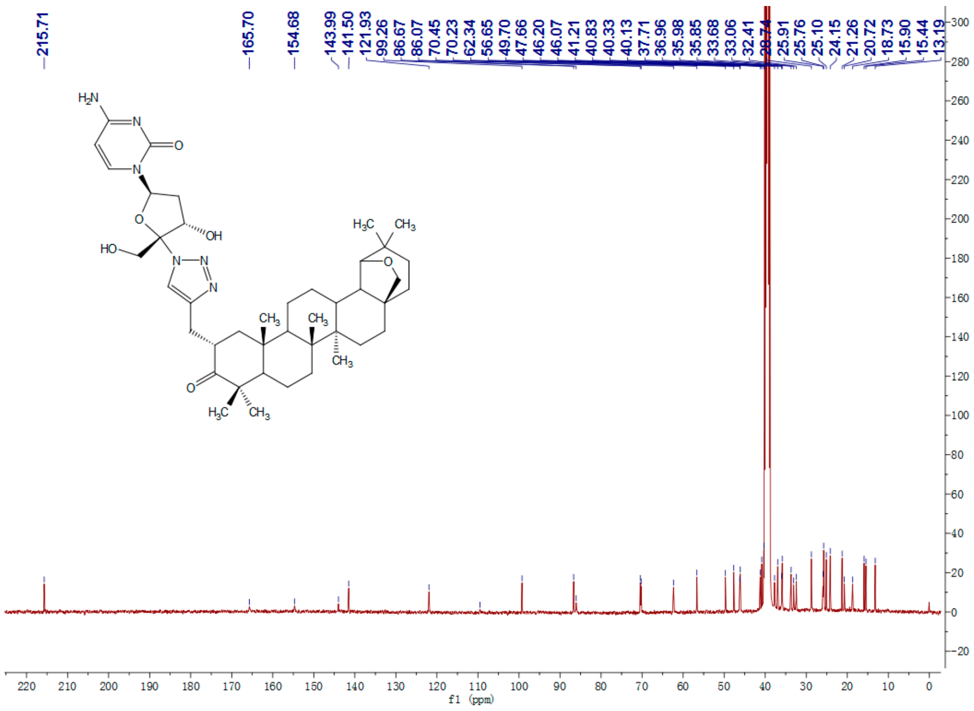

Figure S34 ^{13}C NMR spectrum of compound **9g**

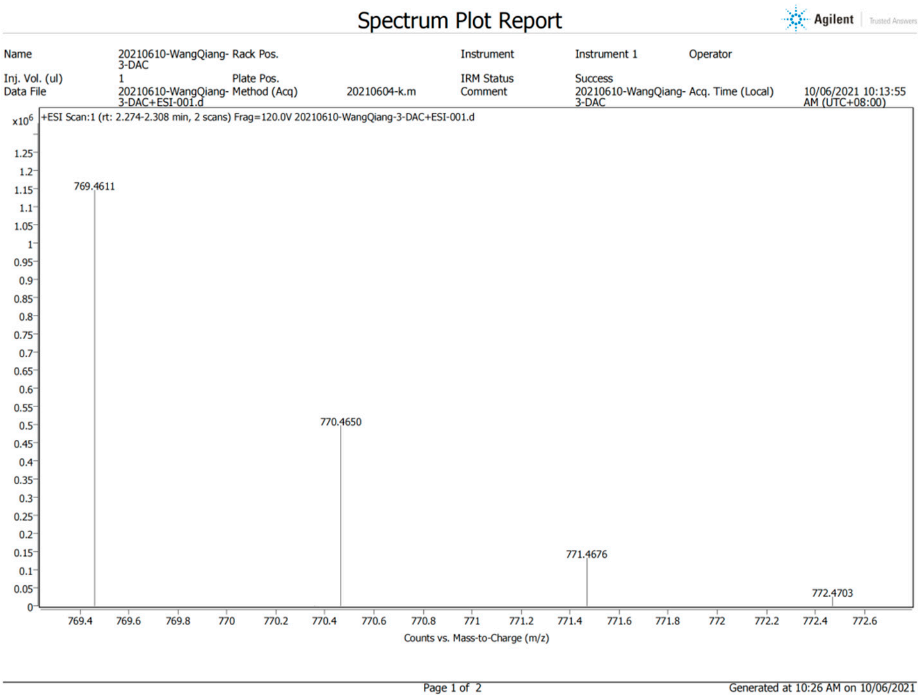

Figure S35 HRMS spectrum of compound **9g**

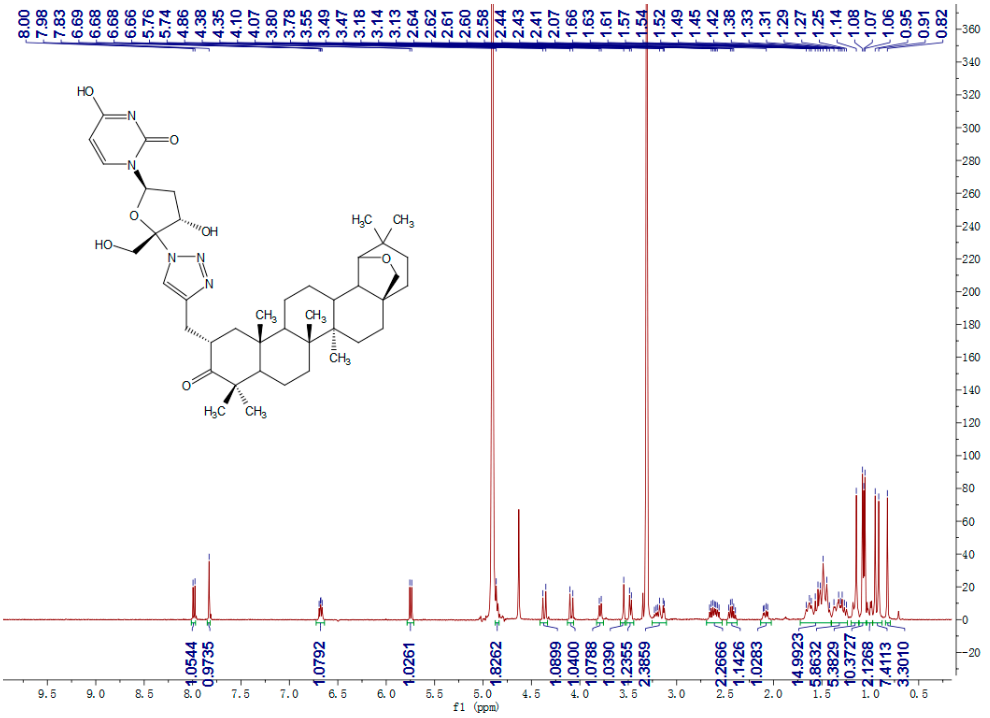

Figure S36 ^{1}H NMR spectrum of compound **9h**

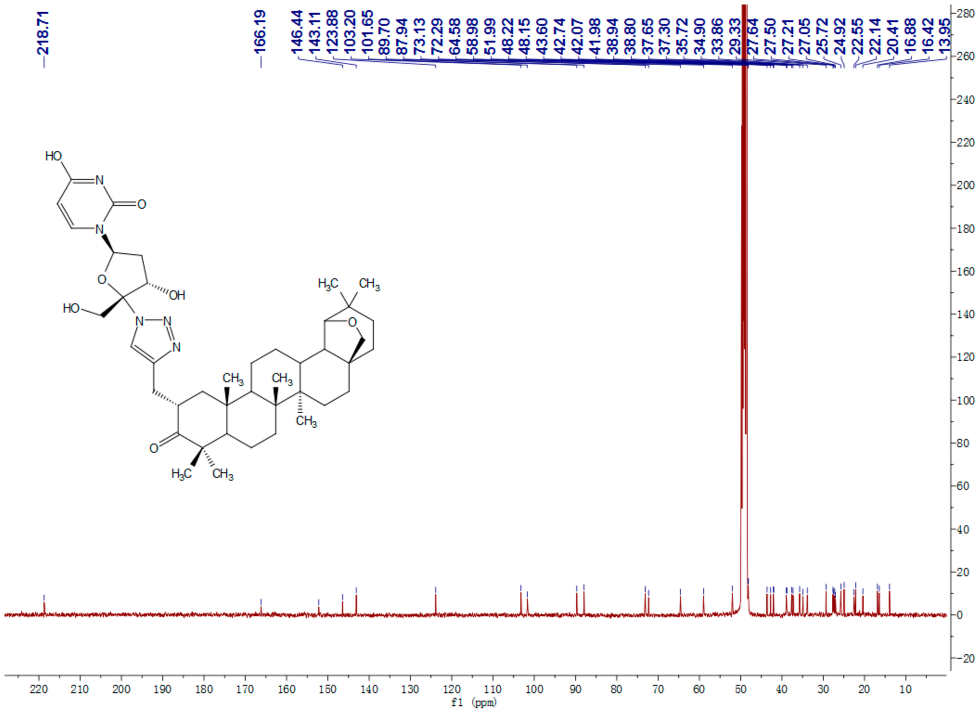

Figure S37 ^{13}C NMR spectrum of compound **9h**

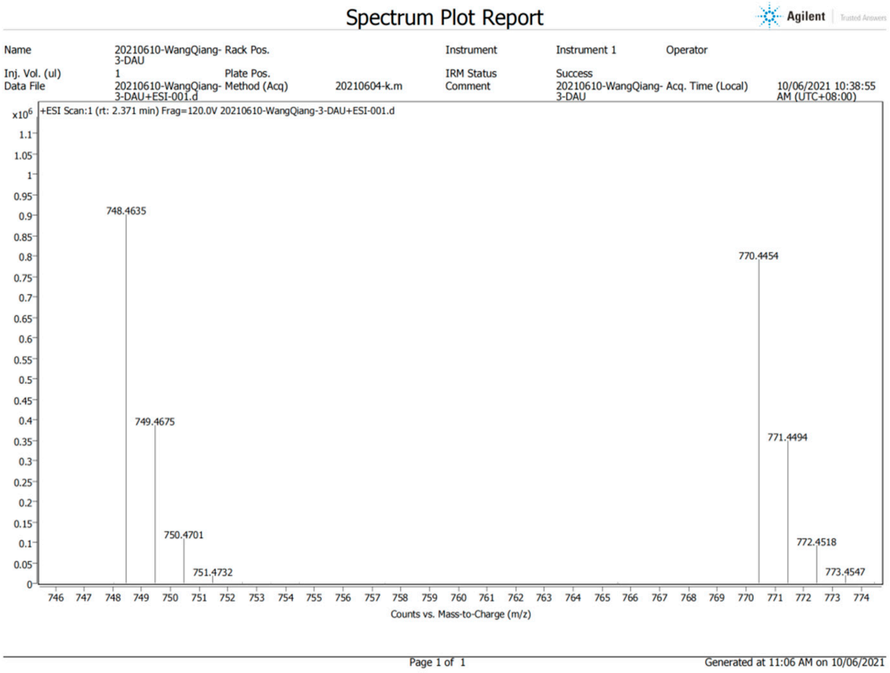

Figure S38 HRMS spectrum of compound **9h**

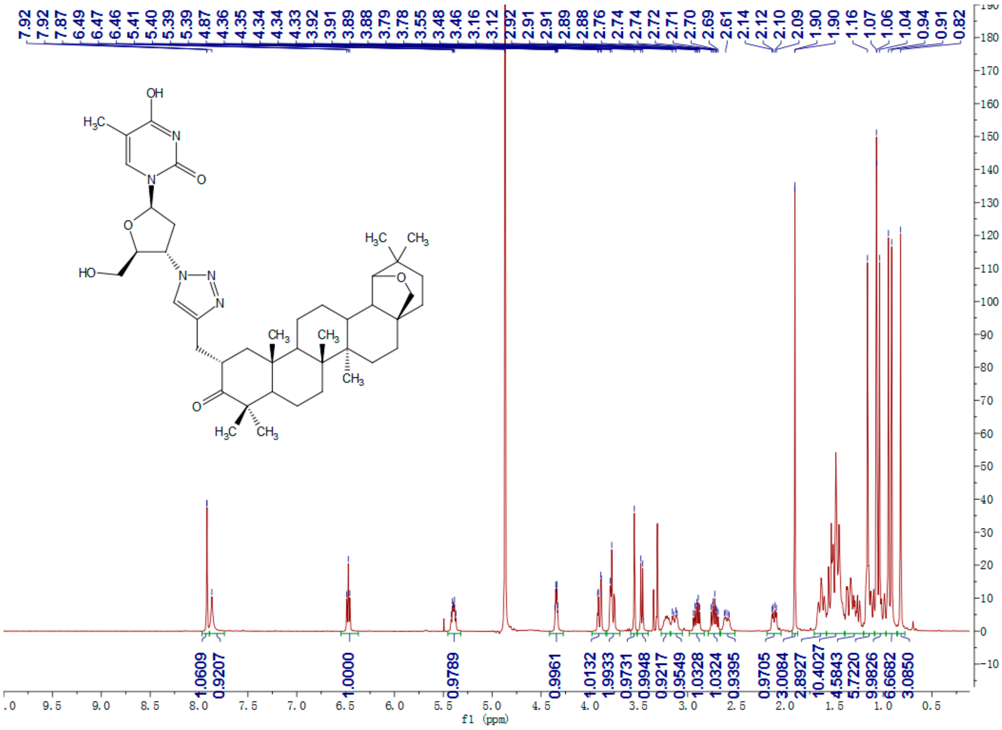

Figure S39 ^{1}H NMR spectrum of compound **9i**

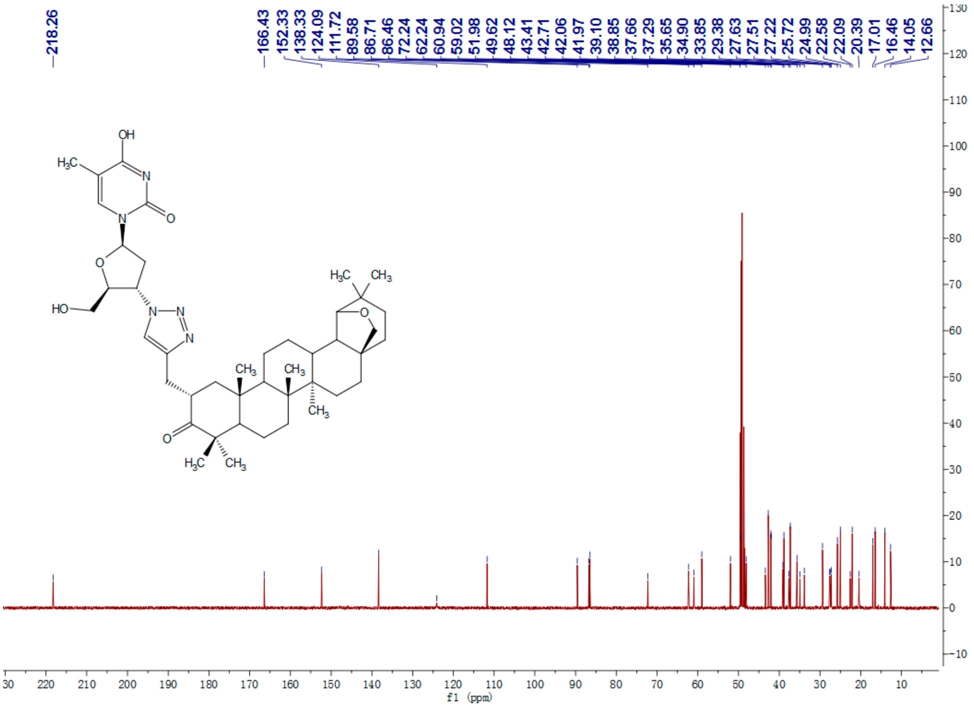

Figure S40 ^{13}C NMR spectrum of compound **9i**

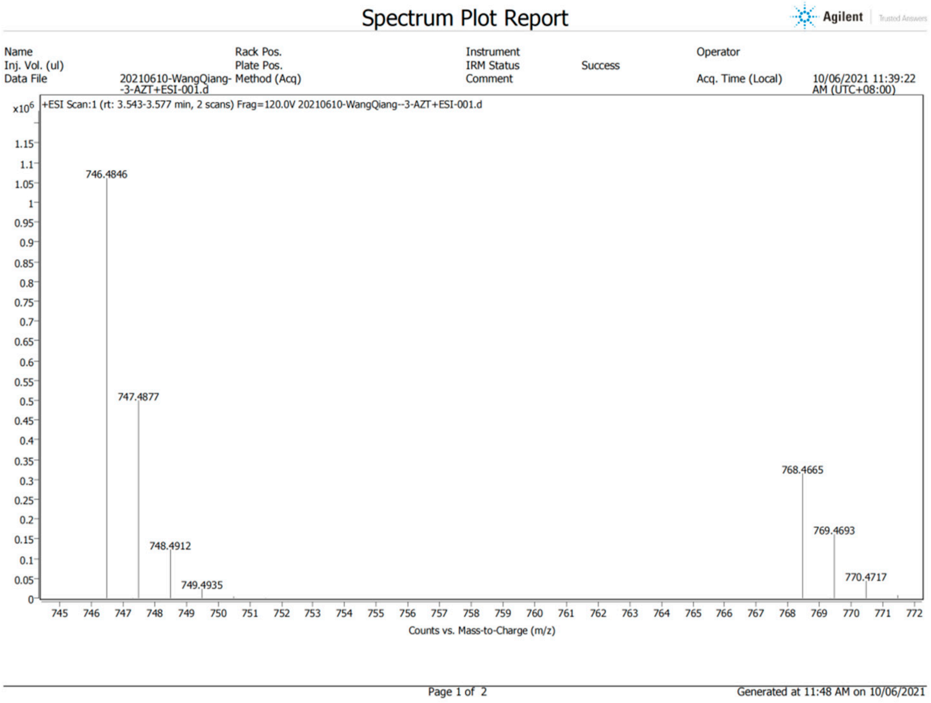

Figure S41 HRMS spectrum of compound **9i**

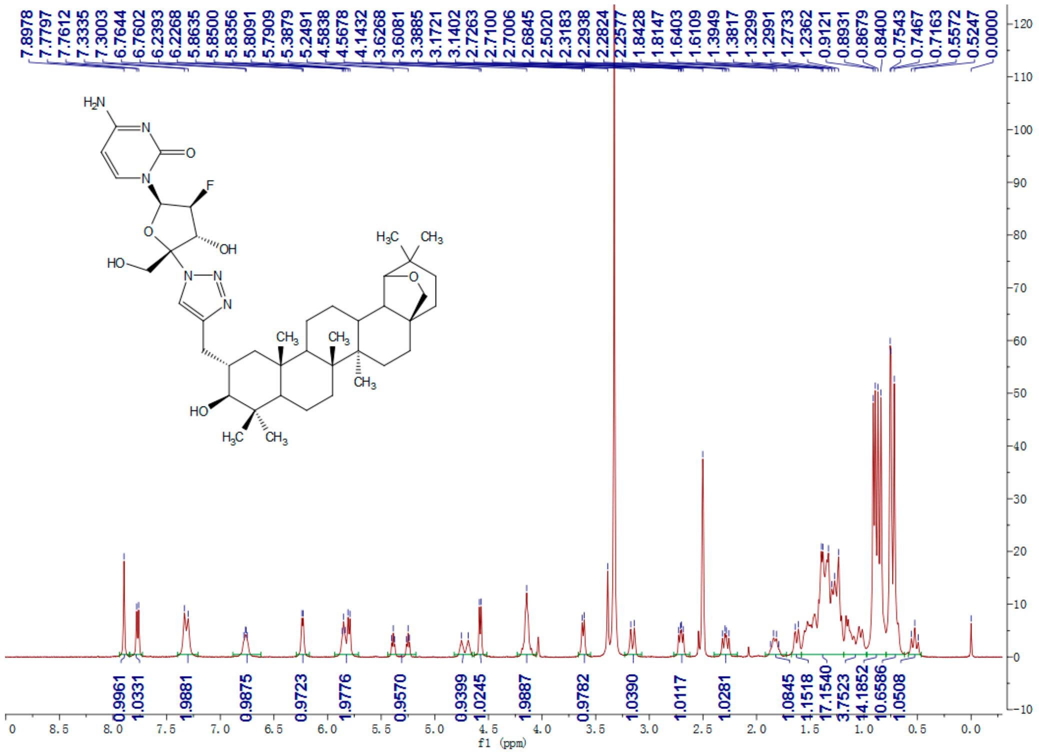

Figure S42 ^{1}H NMR spectrum of compound **10a**

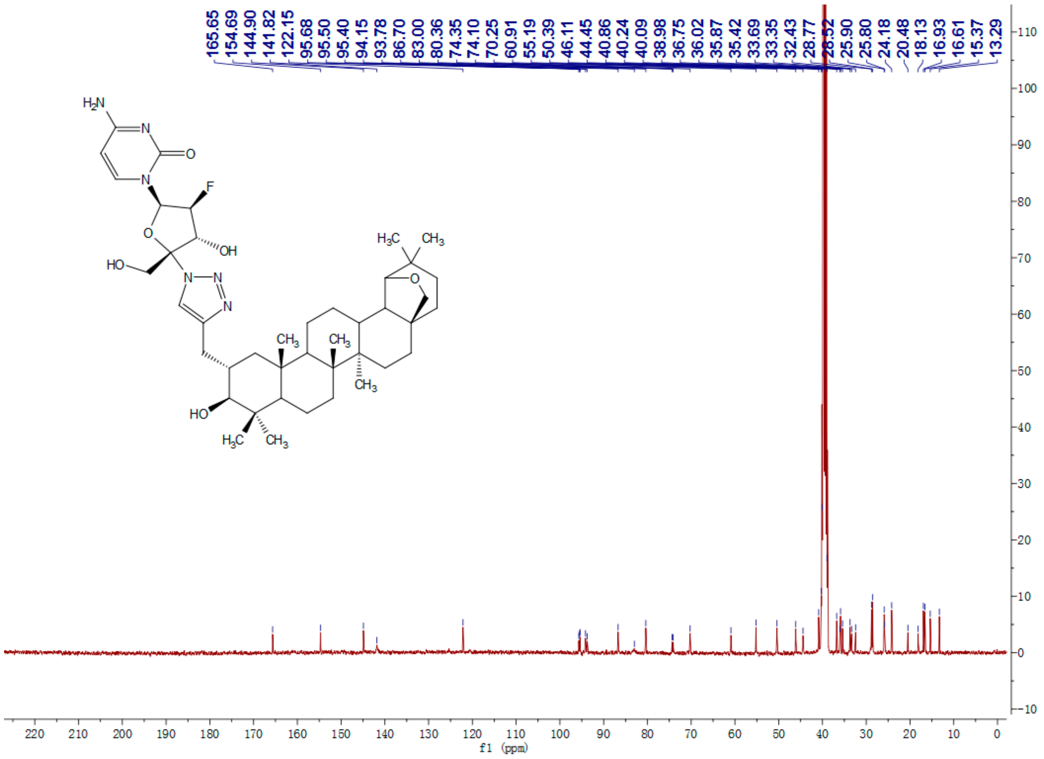

Figure S43 ^{13}C NMR spectrum of compound **10a**

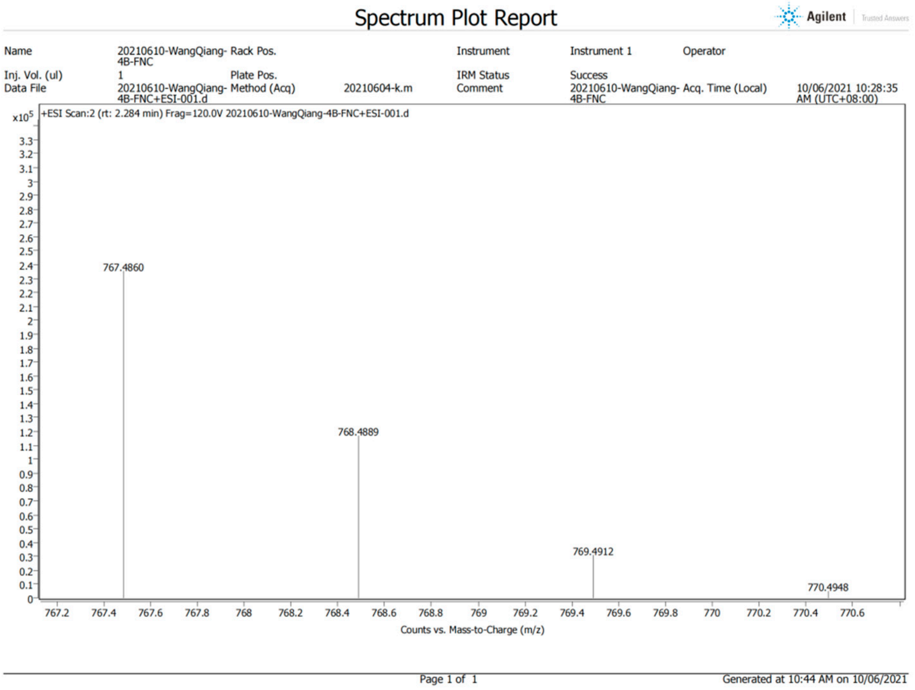

Figure S44 HRMS spectrum of compound **10a**

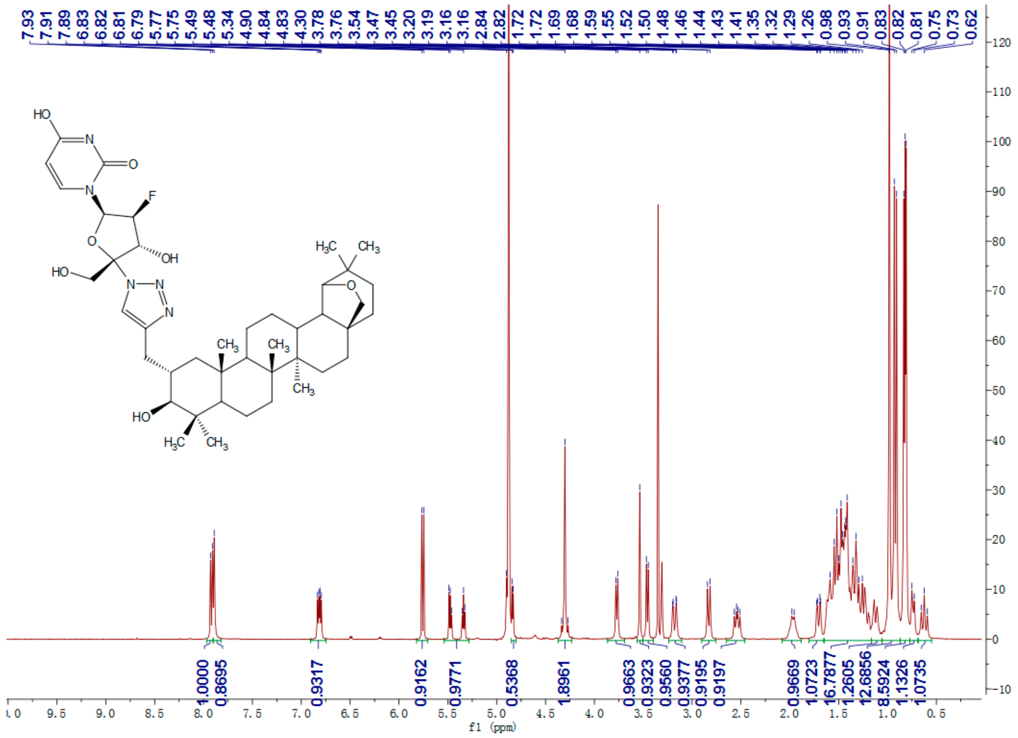

Figure S45 ^{1}H NMR spectrum of compound **10b**

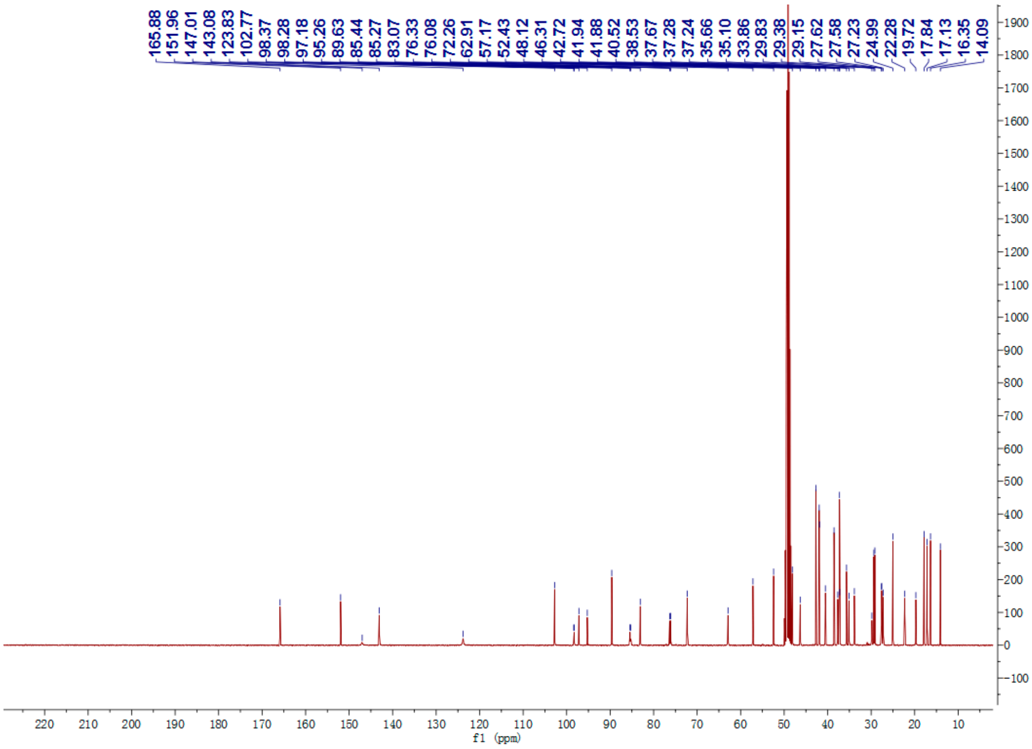

Figure S46 ^{13}C NMR spectrum of compound **10b**

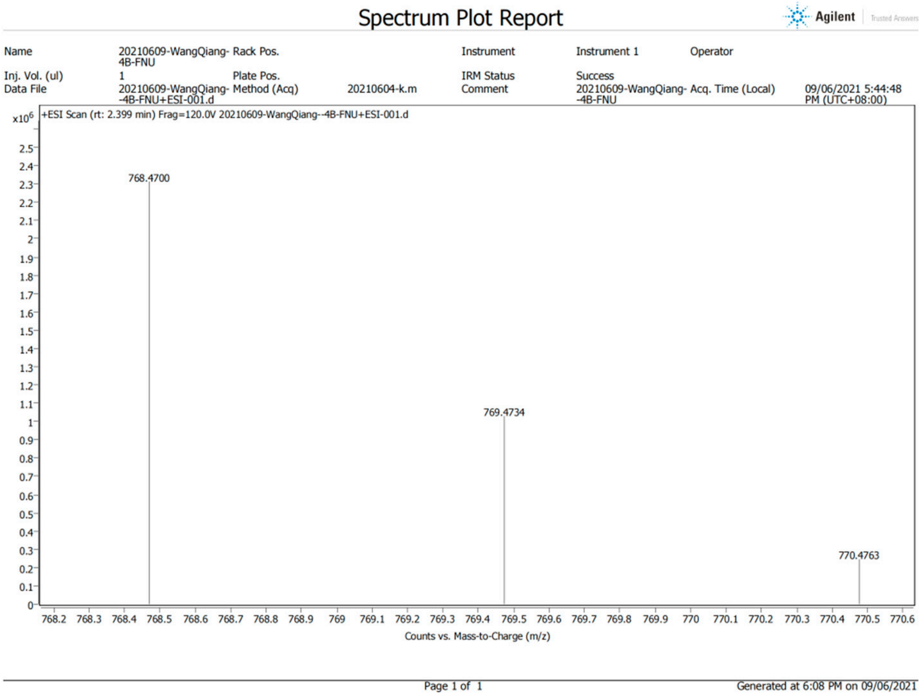

Figure S47 HRMS spectrum of compound **10b**

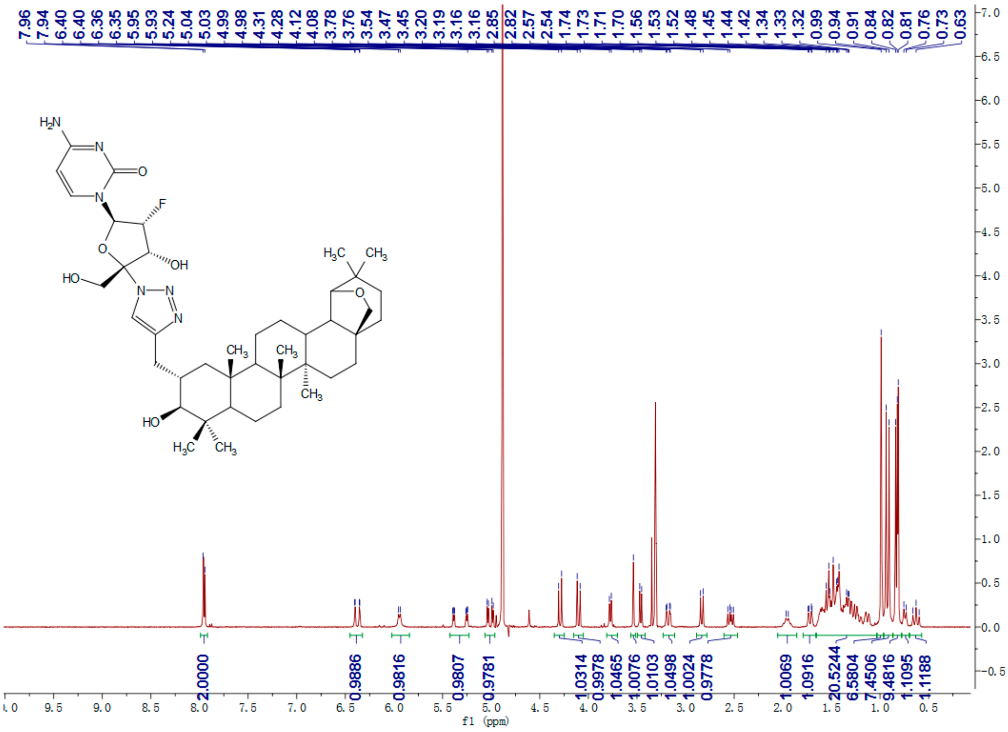

Figure S48 ^{1}H NMR spectrum of compound **10c**

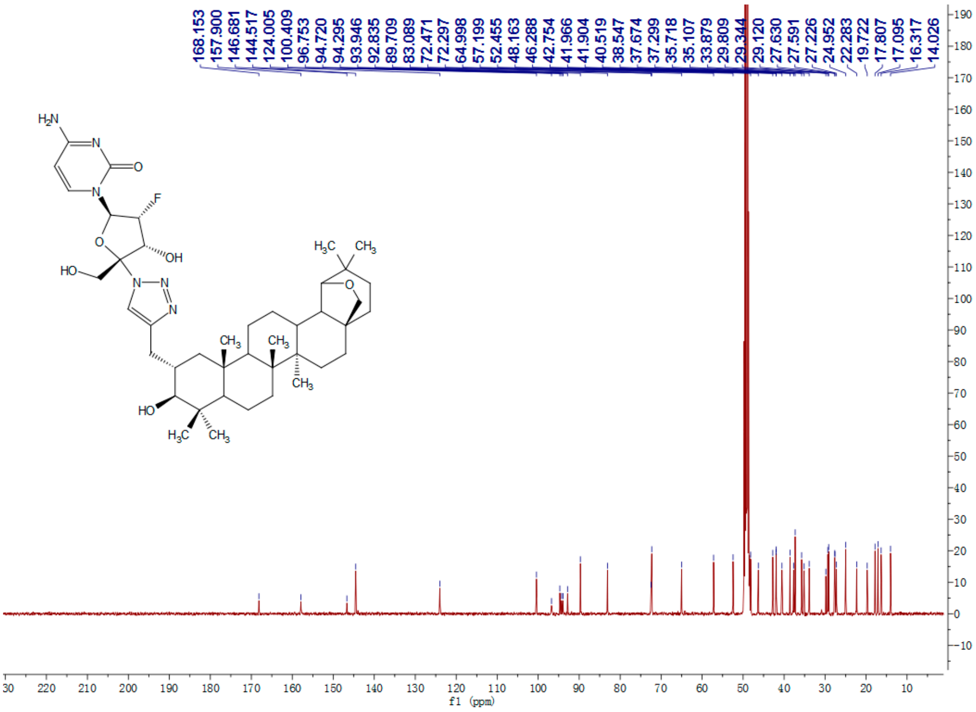

Figure S49 ^{13}C NMR spectrum of compound **10c**

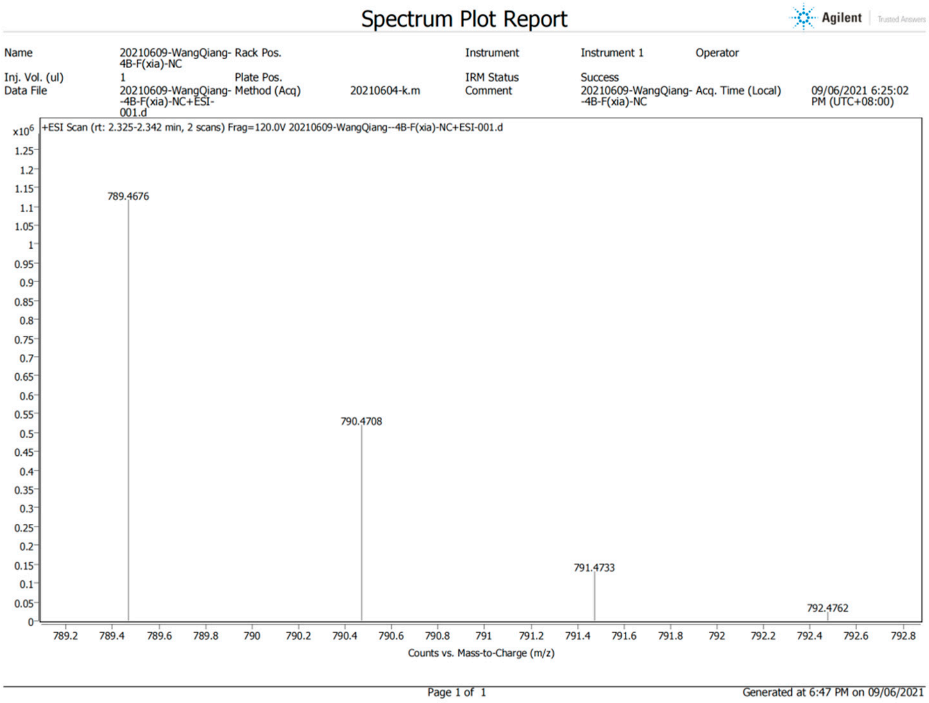

Figure S50 HRMS spectrum of compound **10c**

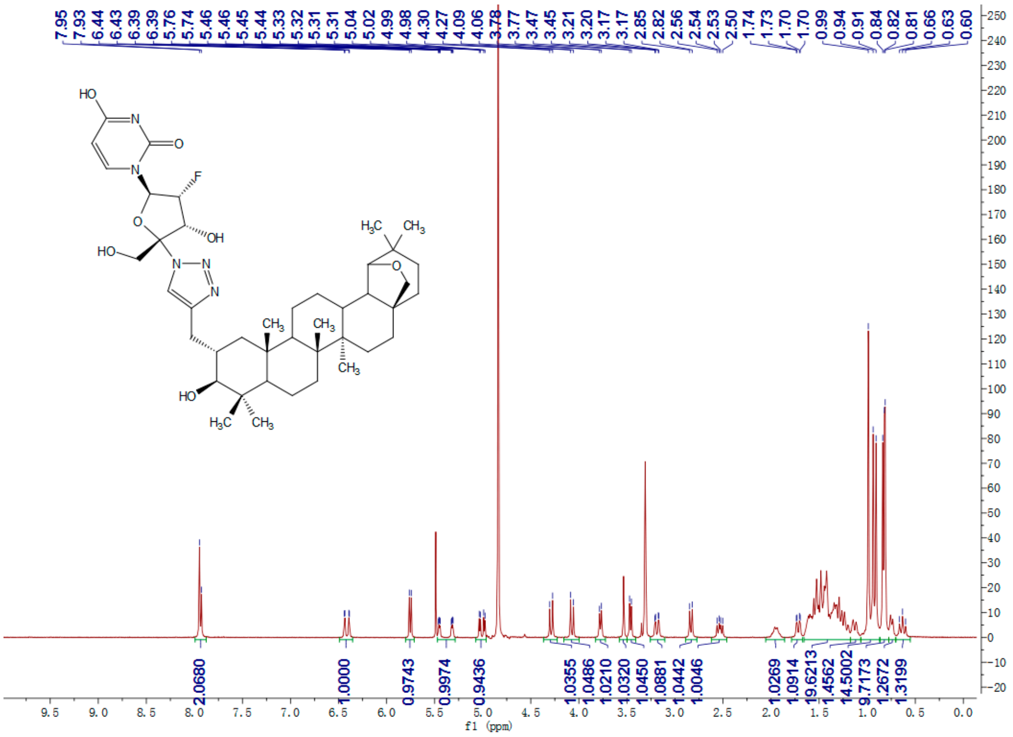

Figure S51 ^{1}H NMR spectrum of compound **10d**

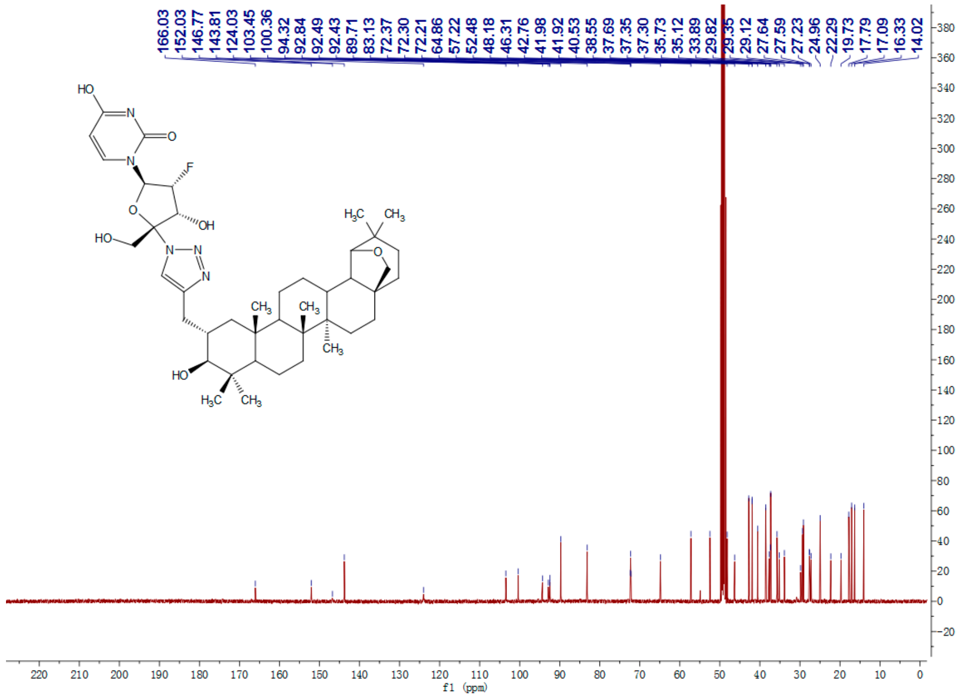

Figure S52 ^{13}C NMR spectrum of compound **10d**

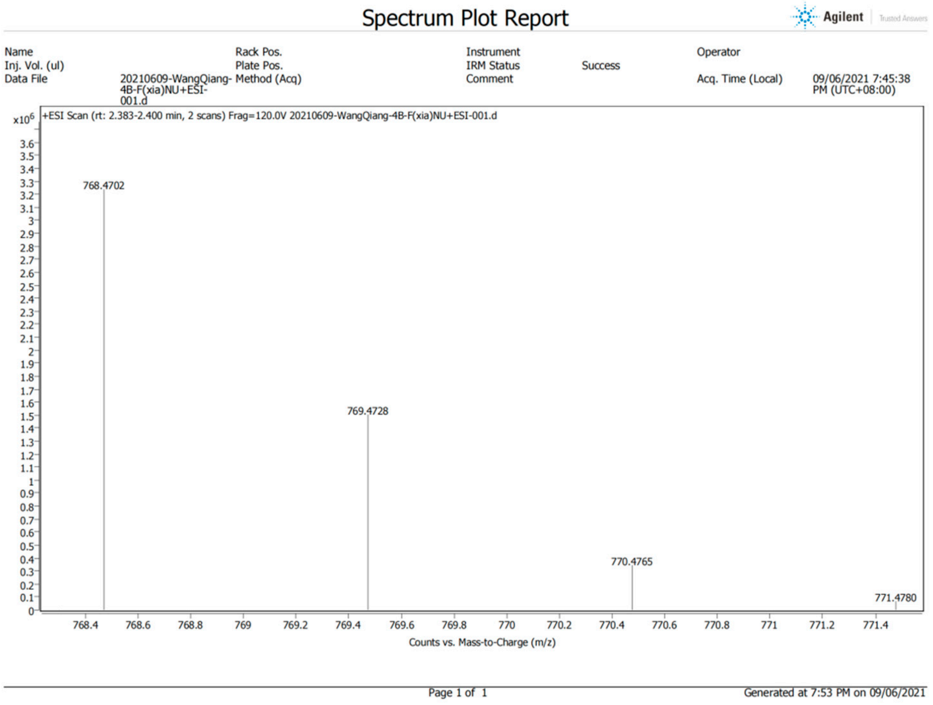

Figure S53 HRMS spectrum of compound **10d**

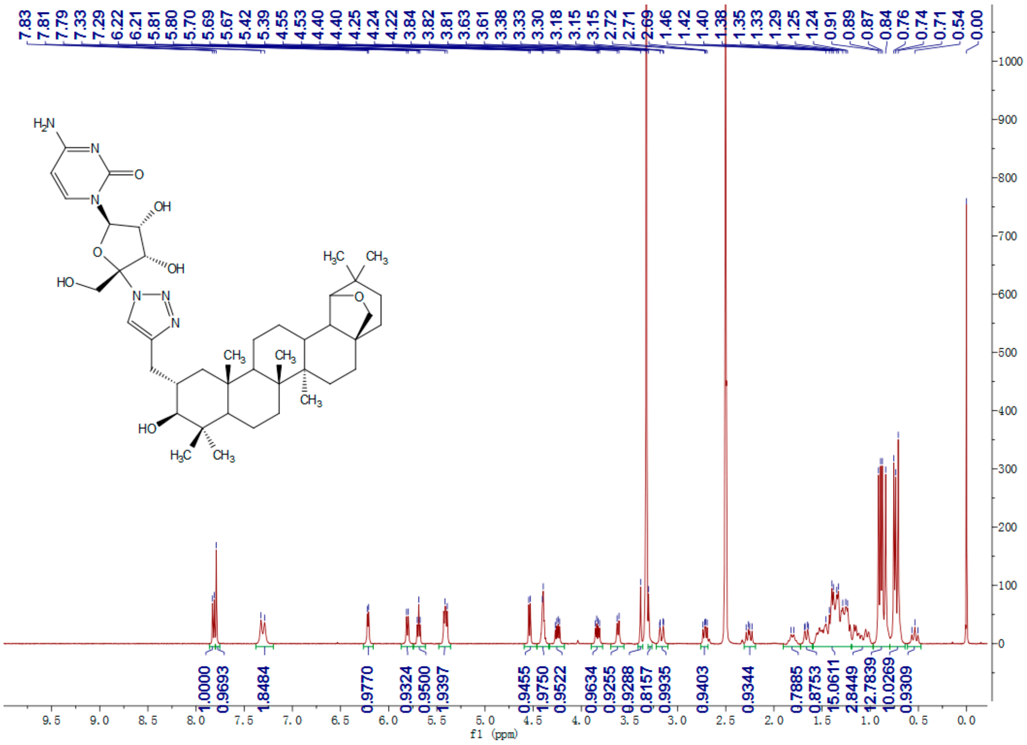

Figure S54 ^{1}H NMR spectrum of compound **10e**

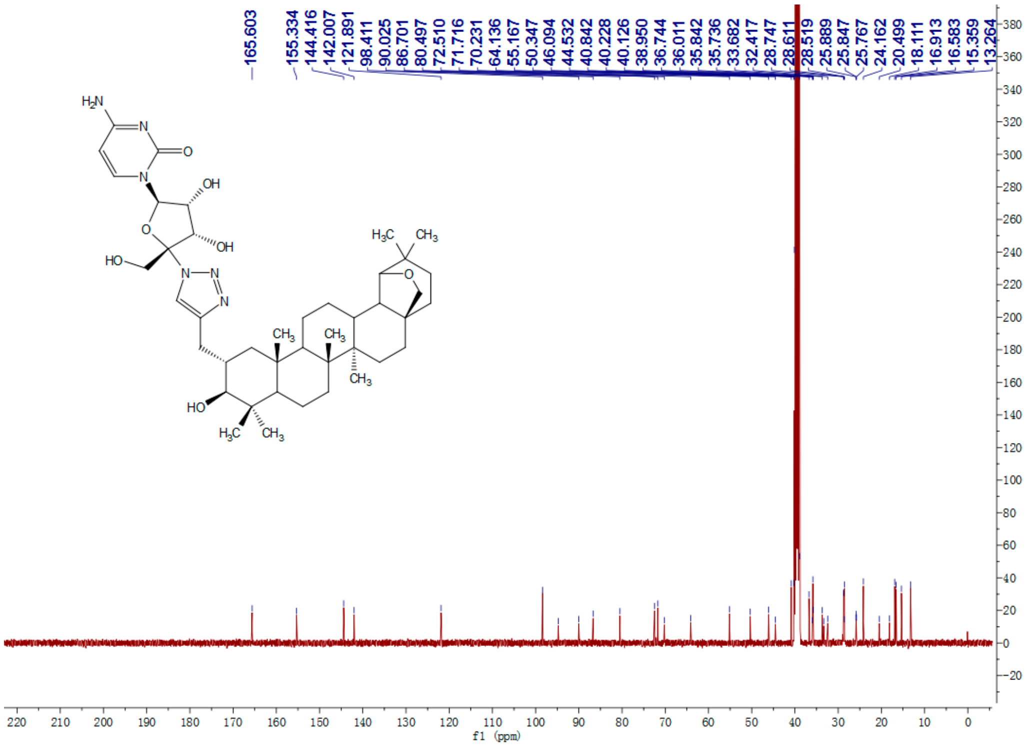

Figure S55 ^{13}C NMR spectrum of compound **10e**

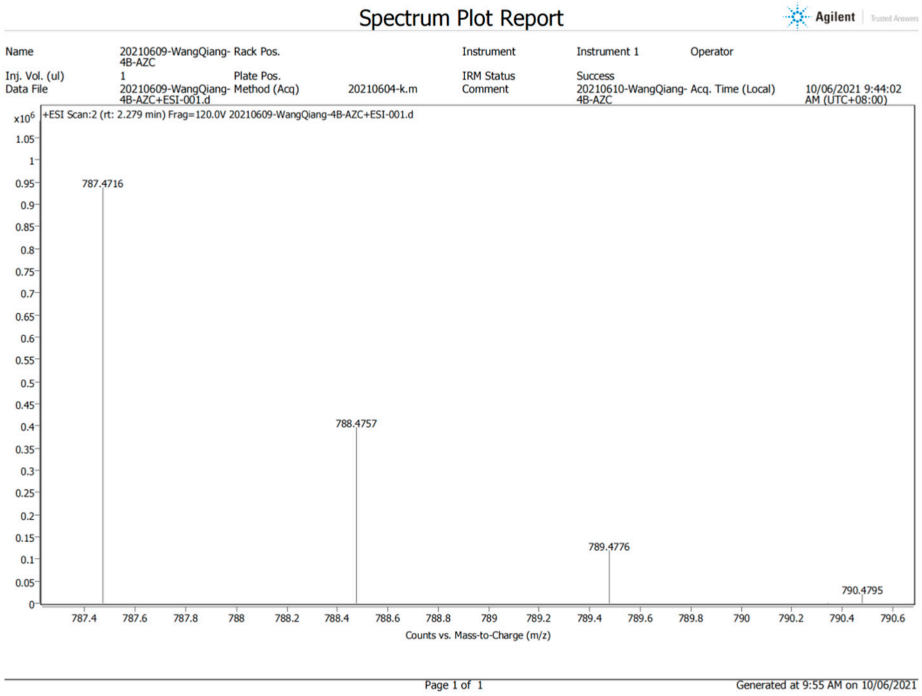

Figure S56 HRMS spectrum of compound **10e**

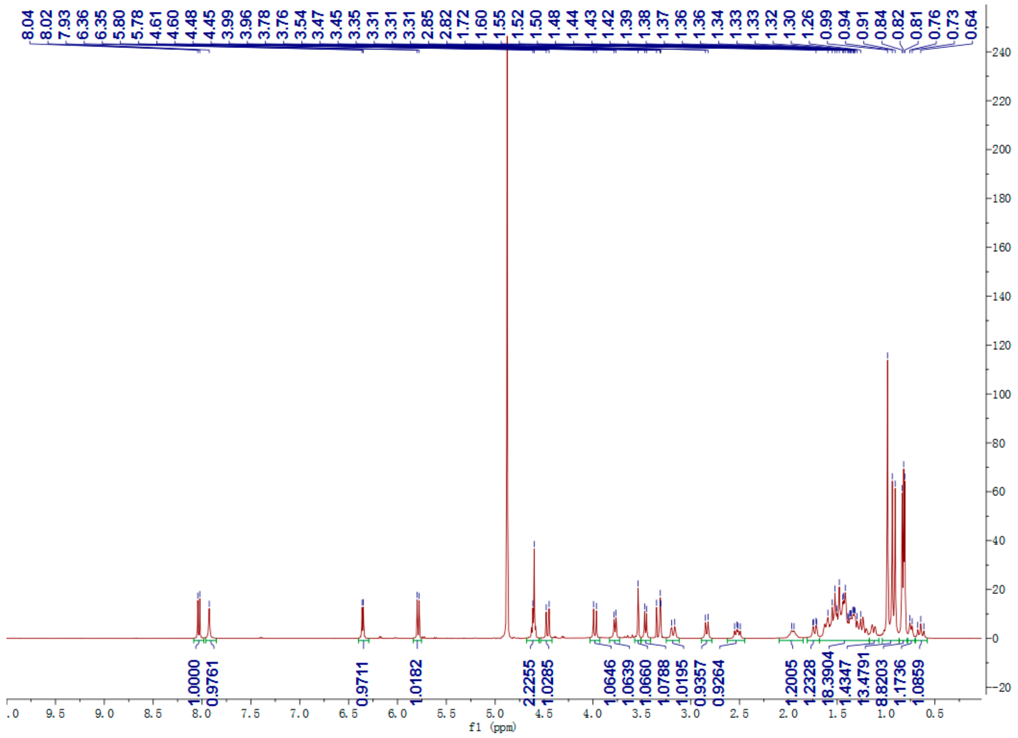

Figure S57 ^{1}H NMR spectrum of compound **10f**

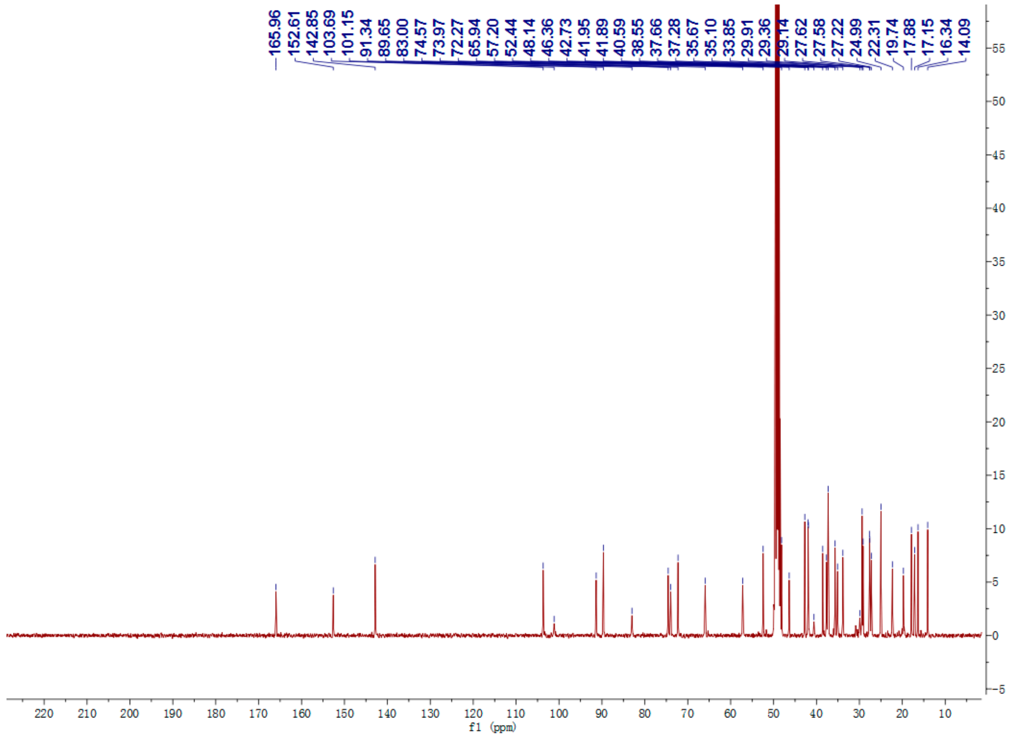

Figure S58 ^{13}C NMR spectrum of compound **10f**

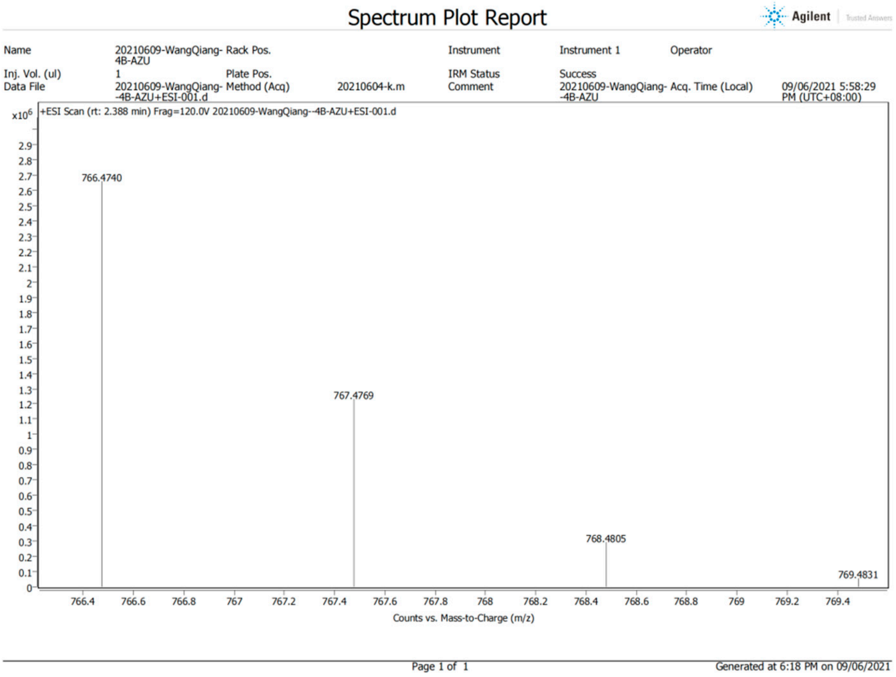

Figure S59 HRMS spectrum of compound **10f**

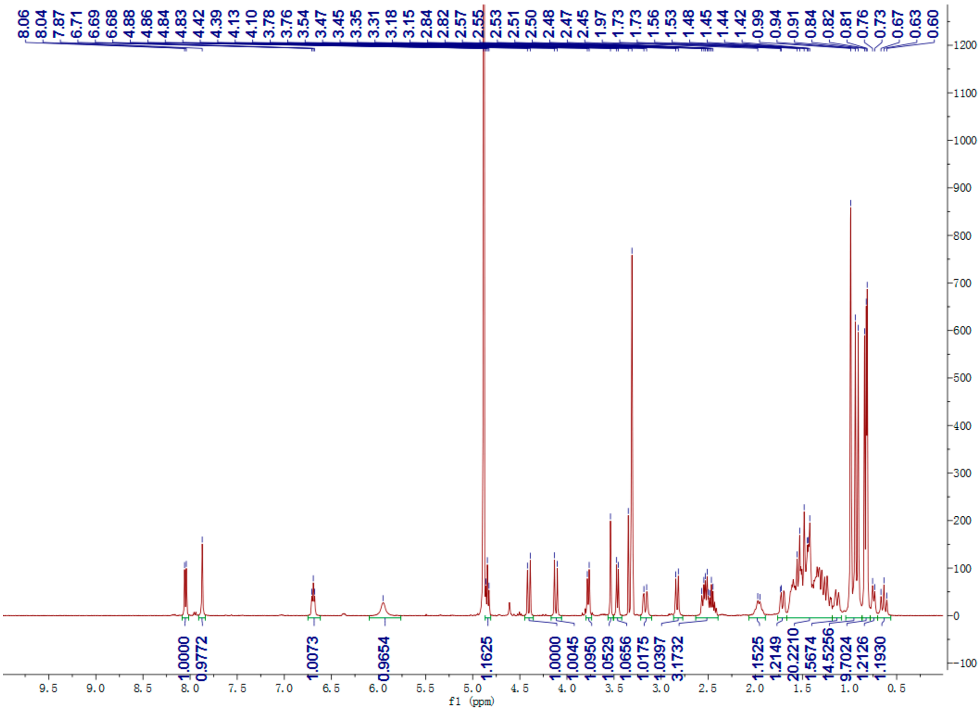

Figure S60 ^{1}H NMR spectrum of compound **10g**

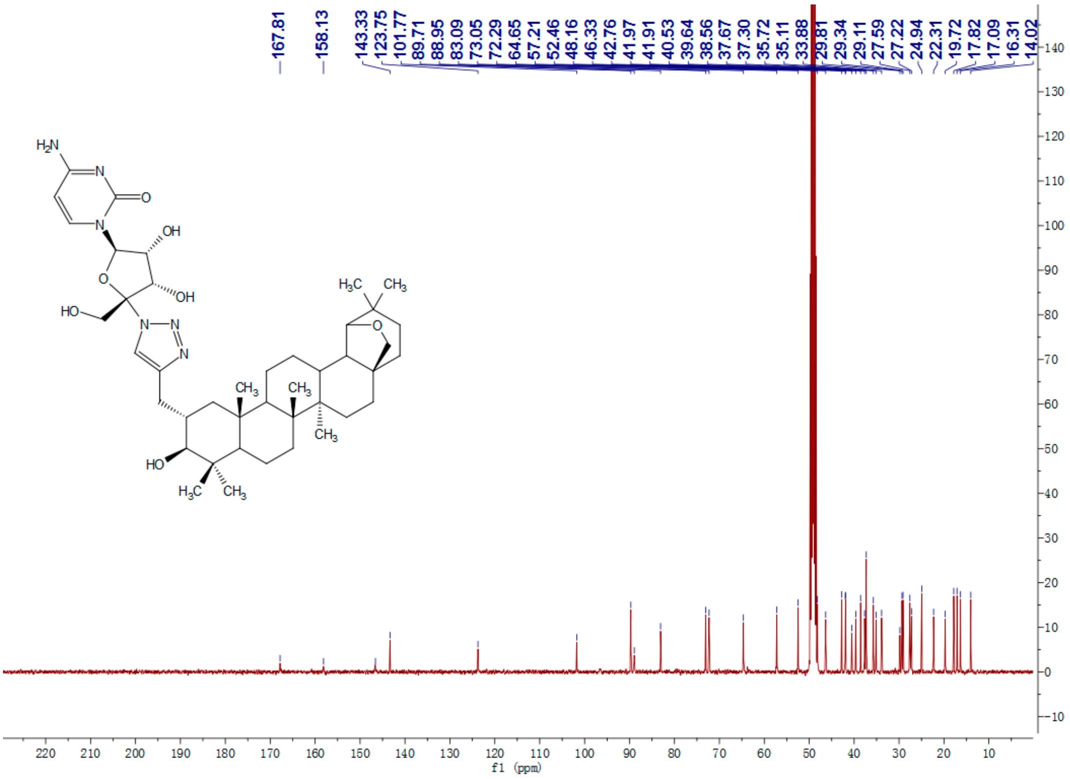

Figure S61 ^{13}C NMR spectrum of compound **10g**

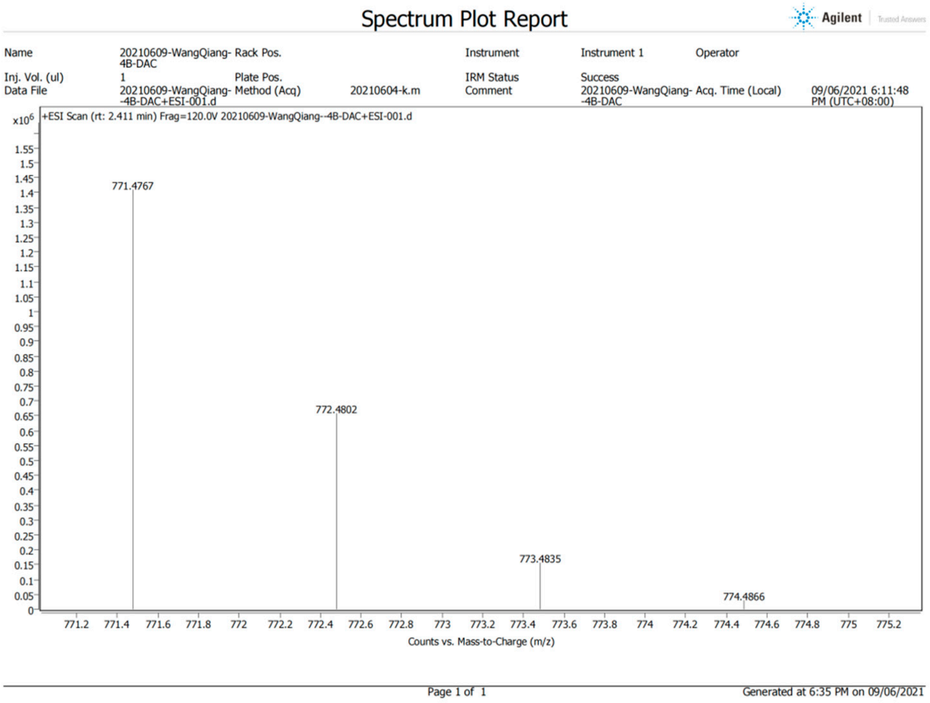

Figure S62 HRMS spectrum of compound **10g**

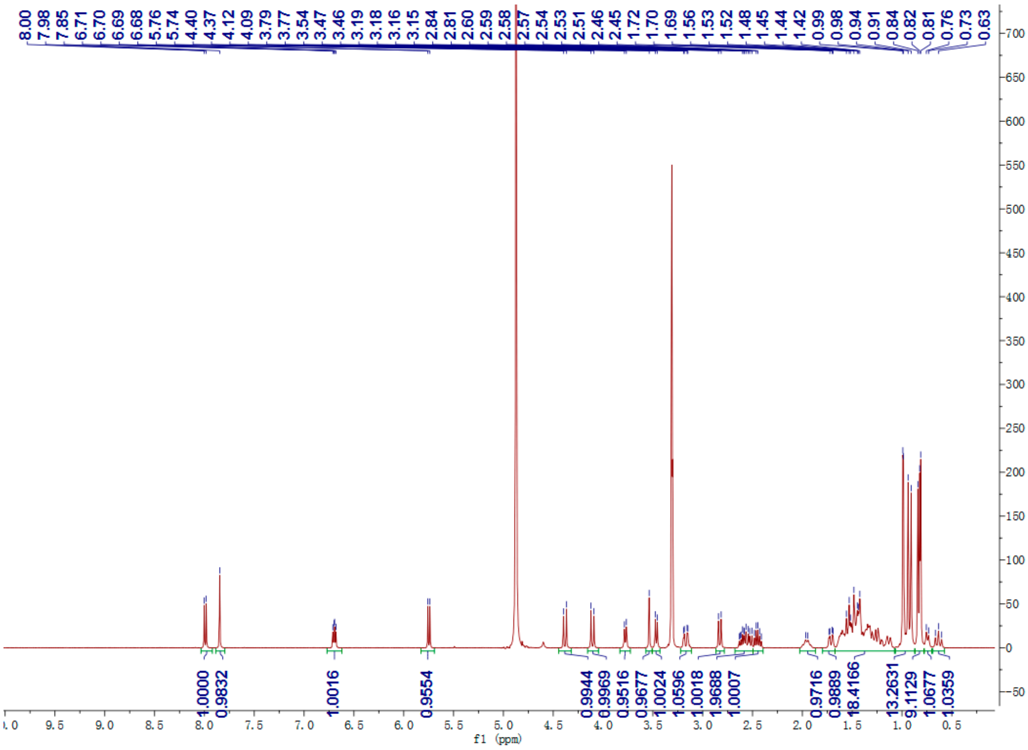

Figure S63 ^{1}H NMR spectrum of compound **10h**

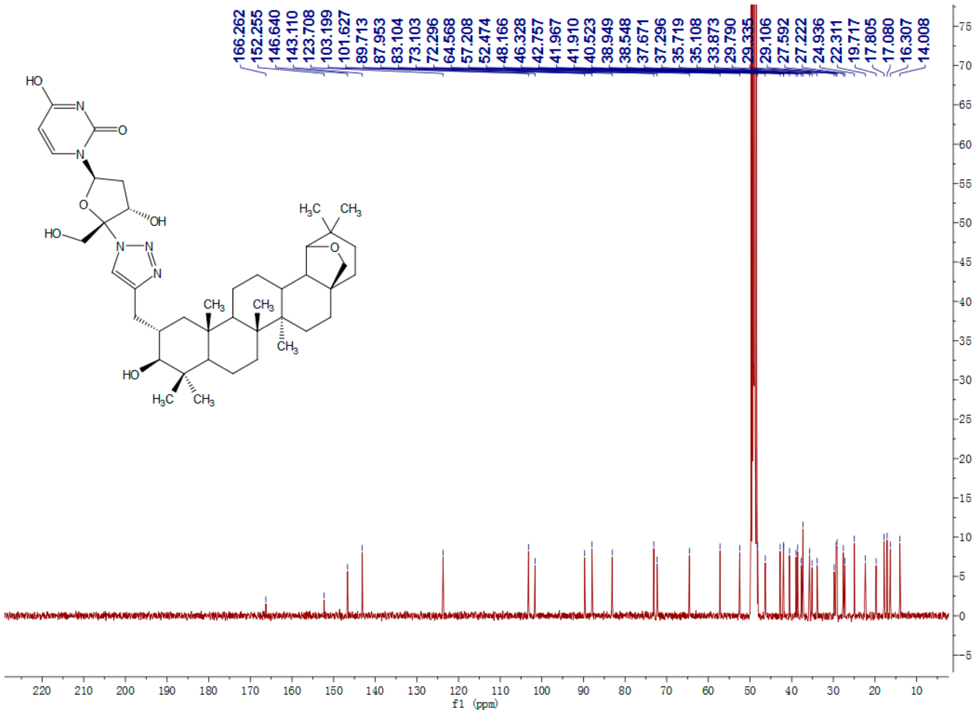

Figure S64 ^{13}C NMR spectrum of compound **10h**

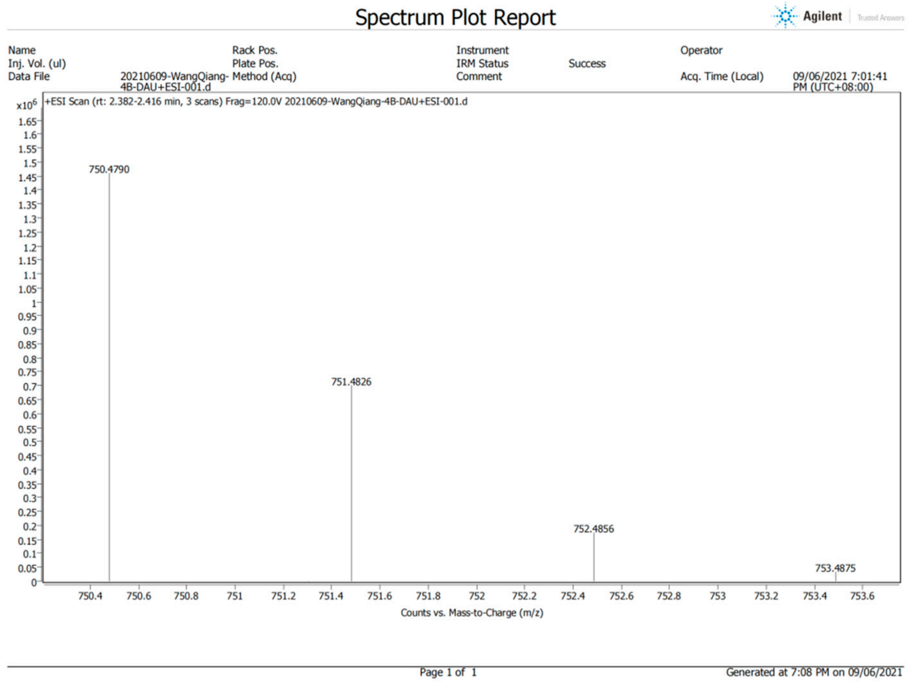

Figure S65　HRMS spectrum of compound **10h**

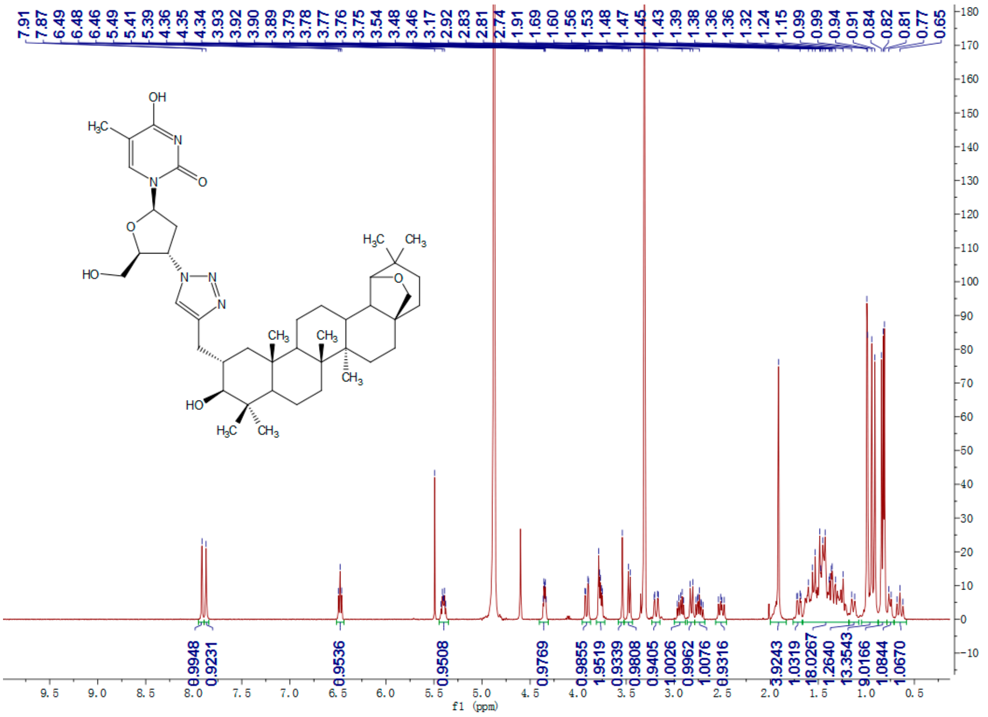

Figure S66　^{1}H NMR spectrum of compound **10i**

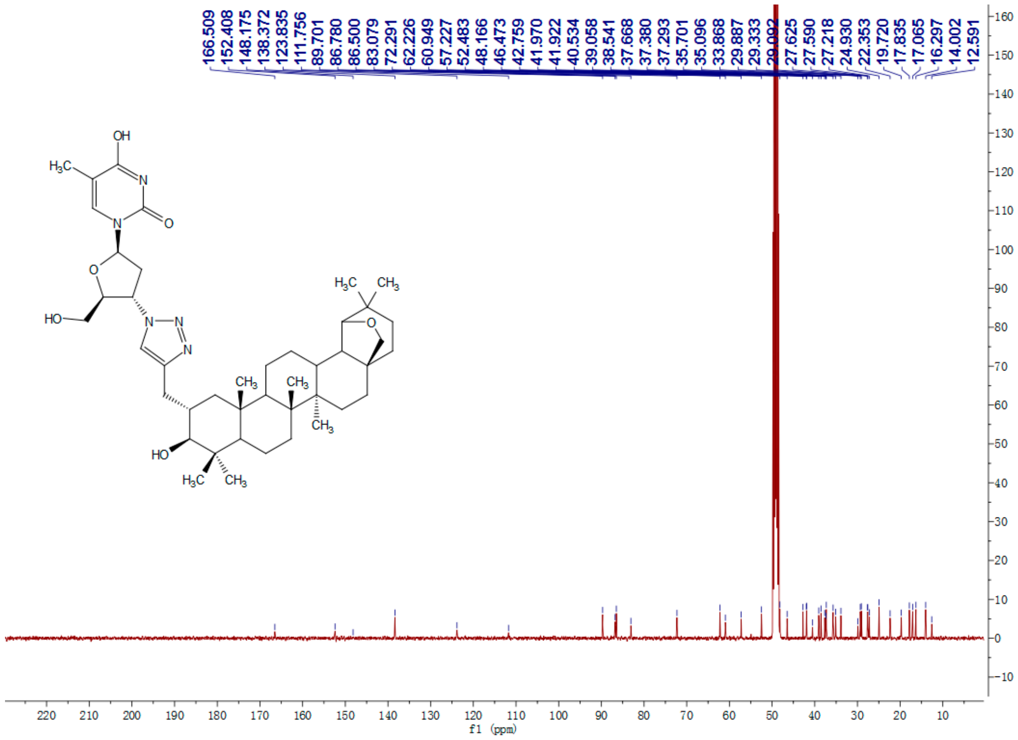

Figure S67 ^{13}C NMR spectrum of compound **10i**

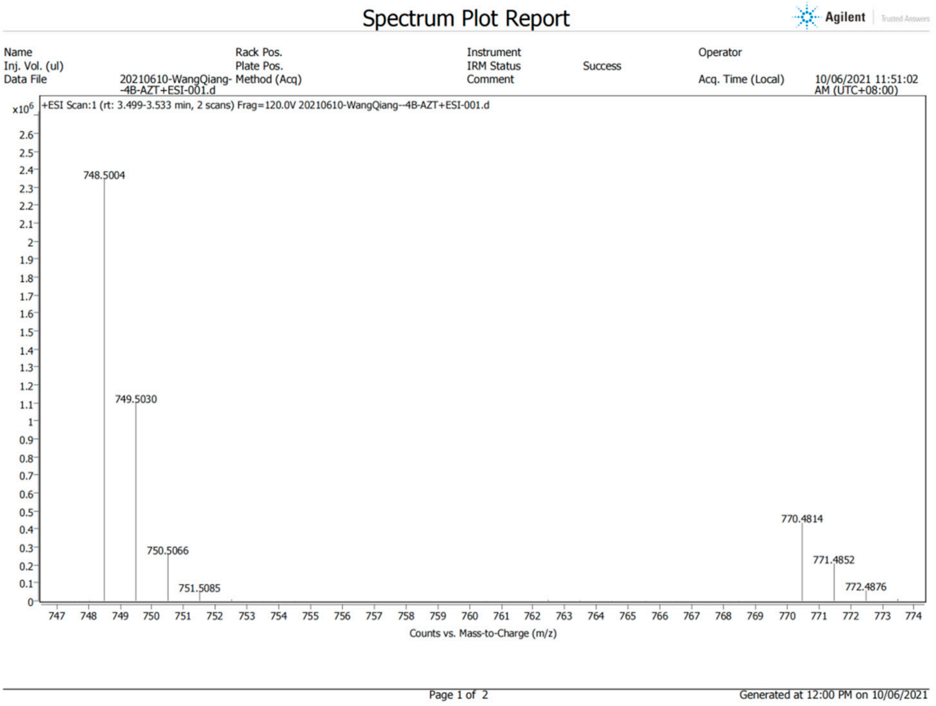

Figure S68 HRMS spectrum of compound **10i**
